# Increased Brain-Age Gap in Young Adults With Psychotic Experiences

**DOI:** 10.1016/j.bpsgos.2025.100643

**Published:** 2025-10-30

**Authors:** Rafael Navarro-González, Pedro Luque-Laguna, Rodrigo de Luis-García, Derek K. Jones, Kate Merritt, Anthony S. David

**Affiliations:** a Universidad de Valladolid, Laboratorio de Procesado de Imagen, Valladolid, Spain; b Headache Unit, Department of Neurology, Hospital Clínico Universitario de Valladolid, Valladolid, Spain; c Cardiff University Brain Research Imaging Centre, Cardiff University, Cardiff, United Kingdom; d Instituto de Investigación Biosanitaria de Valladolid, Valladolid, Spain; e Division of Psychiatry, University College London, London, United Kingdom

**Keywords:** ALSPAC, Brain age, Psychotic experiences, Brain Development, MRI, Longitudinal

## Abstract

**Background:**

Psychotic experiences (hallucinations and delusions: PEs) are linked to structural brain variation, but their relationship to magnetic resonance imaging (MRI)–derived brain age is unclear. We hypothesized that young adults reporting PEs would show an increased brain-age gap (predicted − chronological age) and that this gap would diverge over 10 years.

**Methods:**

A multilayer perceptron (2628 training scans; age 6–50 years; mean absolute error = 4.3 years, *R*^2^ = 0.72) estimated brain age from T1-weighted MRIs in the ALSPAC (Avon Longitudinal Study of Parents and Children). Participants were scanned at around age 20 years (*N* = 245; 124 with PEs) and again at around 30 years (*N* = 279; 69 with PEs); 113 participants contributed both scans. Linear mixed-effects models tested case-control, severity, and time-by-group effects.

**Results:**

At the initial time point, individuals with PEs showed a larger brain-age gap than control individuals (*d* [95% CI] = 0.70 [0.14 to 1.27]; *q* = .029). The brain-age gap showed a trend-level association with PE severity (*d* [95% CI] = 1.32 [0.00 to 2.64]; *q* = .098). At the follow-up, the group difference was nonsignificant (*d* [95% CI] = 0.22 [−0.08 to 0.51]; *q* = .153). No longitudinal case-control divergence reached significance, likely reflecting limited power.

**Conclusions:**

Young adults who report PEs display an older-looking brain in early adulthood, consistent with atypical brain maturation. However, the gap does not clearly widen or contract by age 30. Multimodal, longitudinal cohorts spanning adolescence to midadulthood are needed to map psychosis-related atypical brain maturation.

## Plain Language Summary

Some young people occasionally hear, see, or believe things that others do not—so-called psychotic experiences. We asked whether their brains follow an abnormal developmental path. Using standard MRI scans from the ALSPAC birth cohort, we trained a computer model to estimate brain age and compared 20-year-old individuals with and without these experiences, and we rescanned many of them 10 years later. At age 20, those with psychotic experiences showed subtle signs of altered neurodevelopment, but by age 30, the difference had disappeared. The work suggests early, possibly temporary, changes in brain maturation linked to psychotic experiences.

Brain age, a magnetic resonance imaging (MRI)–based marker, estimates how old a brain looks using machine learning models (1,2). The difference between this estimate and chronological age, known as the brain-age gap, brain-age gap estimate, or brain-predicted age difference (BrainPAD), is considered an age-adjusted index of brain integrity. A positive gap, indicating an older-than-expected brain, has been reported in neurological, psychiatric, and metabolic disorders such as chronic migraine, schizophrenia, and hypertension (3, 4, 5). Furthermore, early-life adversity (6,7) is also related to a positive gap. On the other hand, protective sociological and lifestyle factors such as years of education, regular physical exercise, or practicing meditation have been linked to a negative BrainPAD (8,9).

Brain-age tools broadly show larger gaps in chronic psychoses (5,10); in schizophrenia, the gap is wide, indicating marked decline (11,12). This shift has been linked to chronic symptoms, neuro-inflammation, prolonged stress, and early neurodevelopmental insults (13,14). Crucially, we still do not know whether a similar gap is already observable in young people with psychotic experiences (PEs). PEs, brief hallucination- or delusion-like experiences in otherwise healthy individuals, affect ∼7% of the population ages 16 to 19 years, according to Yates *et al.* (15). Leveraging the ALSPAC-PE (Avon Longitudinal Study of Parents and Children-Psychotic Experiences) dataset, Drakesmith *et al.* (16,17) showed that at age 20, individuals with PEs differed neuroanatomically from their peers without PEs. Specifically, participants with PEs displayed reduced gray matter volume, altered T1 relaxation rates, and widespread white matter microstructural alterations on diffusion tensor imaging (DTI). Consistent with these findings, Merritt *et al.* (18) reported reduced volume in the thalamus and posterior cingulate in individuals with PEs. Such patterns hint at an atypical maturation course in PEs.

Mapping the PE BrainPAD could refine theory, flag early psychosis risk, guide prevention, and track brain changes (19). Building on this premise, we tested the cross-sectional and longitudinal BrainPADs in a large young adult cohort with PEs. Training a brain-age model on T1-weighted MR images and applying it to the ALSPAC-PE cohort, we tested 3 hypotheses. First, we hypothesized that PE-positive young adults scanned at age 20 years (*n* = 124) would show a larger BrainPAD than PE-negative control adults (*n* = 121) and that this group difference would still be evident at 30 years (PE-positive = 69; control = 210). Second, we hypothesized that BrainPAD would increase with PE severity at both ages. Finally, we hypothesized that the 10-year within-subjects change in the BrainPAD would diverge by PE status, indicating persistence, widening, or normalization of the gap. This design combines age-specific cross-sectional contrasts with a decade-long longitudinal analysis, allowing full assessment of brain-age gap trajectories in subclinical PEs.

## Methods and Materials

### Application Dataset

The application dataset for our study comes from the ALSPAC-PE cohort (16,20). This study is nested within the ALSPAC cohort, a population-based study aimed at determining contributing factors in children’s health and development (21, 22, 23). The cohort is described in more depth in Supplement, section ALSPAC Study Population. The longitudinal cohort comprises 3 overlapping subsamples: age 20 (ALSPAC-20 MRI-I), age 30 (ALSPAC-30 MRI-II), and a longitudinal age 20 to age 30 sample (participants with MRI data available at both age 20 and 30 years). Different scanners were used to acquire MRI data at age 20 and 30. At age 18, study children were sent fair processing materials describing ALSPAC’s intended use of their health and administrative records and were given clear means to consent or object via a written form. Data were not extracted for participants who objected or who were not sent fair processing materials. Study data were collected and managed using REDCap tools hosted at the University of Bristol (24). REDCap is a secure, web-based software platform designed to support data capture for research studies. Note that the study website contains details of all the data available through a fully searchable data dictionary and variable search tool (https://www.bristol.ac.uk/alspac/researchers/our-data/).

Ethical approval for the study was obtained from the ALSPAC Law and Ethics Committee and the local research ethics committees (National Health Service REC:10/H1010/70, listed at http:/www.bristol.ac.uk/alspac/researchers/research-ethics/). Informed consent for the use of data collected via questionnaires and clinics was obtained from participants following the recommendation of the ALSPAC Ethics and Law Committee at the time.

#### ALSPAC-20 MRI-I

**Figure 1:**
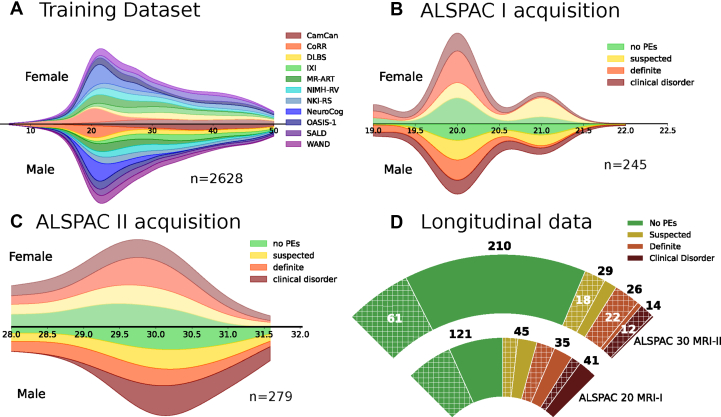
The demographic distribution across different datasets and acquisition time points. **(A)** Age and sex distribution of the training dataset (*n* = 2628) across multiple cohorts. **(B)** Age and sex distribution of participants in the ALSPAC (Avon Longitudinal Study of Parents and Children) 20 magnetic resonance imaging (MRI) I acquisition (*n* = 245), categorized by psychotic experiences (PEs) (no PEs, suspected, definite, and clinical disorder). **(C)** ALSPAC-30 MRI-II acquisition (*n* = 279) with the same PE categories. **(D)** Data distribution across the cross-sectional and longitudinal subsamples (participants scanned at both waves, *n* = 113). The inner arc represents ALSPAC-20 MRI-I; the outer arc represent ALSPAC-30 MRI-II. Hatched wedges highlight individuals present at both time points, with white numerals indicating the overlap count in each category. Bold black numerals outside the arcs show the total per Psychotic-like Symptoms (PLIKS)-18 category at each wave. CamCAN, Cambridge Centre for Ageing and Neuroscience; CoRR, Consortium for Reliability and Reproducibility; DLBS, Dallas Lifespan Brain Study; IXI, Information eXtraction from Images; MR-ART, Movement-Related Artefacts; NeuroCog, Neurocognitive Aging Data Release; NIMH-RV, National Institute of Mental Health – Research Volunteer; NKI-RS, Nathan Kline Institute – Rockland Sample; OASIS-1, Open Access Series of Imaging Studies; SALD, Southwest University Adult Lifespan Dataset; WAND, Welsh Advanced Neuroimaging Database.

A subset of 4320 individuals of the ALSPAC cohort was reassessed for PEs at age 17/18 using the Psychotic-like Symptoms (PLIKS) interview (25,26) conducted by trained psychologists. The PLIKS inventory is further explained in the Supplement. Individuals with PEs were categorized in a stratified manner as suspected (*n* = 41, PLIKS-18 = 1), definite (*n* = 45, PLIKS-18 = 2), and clinical disorder (definite PEs with functional decline or help seeking; *n* = 35, PLIKS-18 = 3) (27). Individuals with 1 or more PEs were invited for brain imaging. Of the 249 participants who were scanned, 4 were excluded due to quality issues. A total of 121 individuals with PEs and 124 randomly selected control individuals, who had completed the same assessments but did not report PEs, were selected. All participants were approximately age 20 years at the time of scanning. See Table 1 and Figure 1 for more details.

**Table 1:** Summary of Demographic Data Across the Cross-Sectional Test Groups

Dataset	*n*	Age, Years, Mean (SD) [Range]	Sex, Female/Male, *n*	Statistics
ALSPAC-20 MRI-I				
Control, No PEs	124	20.18 (0.63) [19–22]	75/49	Mann-Whitney *U*, *p* = .385 Sex χ^2^, *p* = .233
PEs	121	20.10 (0.60) [19–21]	83/38	
Suspected, PLIKS-18 = 1	41	20.49 (0.51) [20–21]	27/14	
Definite, PLIKS-18 = 2	45	19.84 (0.48) [19–21]	29/16	
Clinical disorder, PLIKS-18 = 3	35	19.97 (0.62) [19–21]	27/8	
Total	245	20.14 (0.61) [19–22]	158/87	
ALSPAC-30 MRI-II				
Control, No PEs	210	29.47 (1.16) [27–31]	87/123	Mann-Whitney *U*, *p* = .928 Sex χ^2^, *p* = .004
PEs	69	29.47 (1.14) [27–32]	43/26	
Suspected, PLIKS-18 = 1	29	29.45 (1.23) [27–32]	15/14	
Definite, PLIKS-18 = 2	26	29.44 (1.02) [27–31]	17/9	
Clinical disorder, PLIKS-18 = 3	14	29.55 (1.23) [27–31]	11/3	
Total	279	29.47 (1.15) [27–32]	130/149	

The data are divided by cohort and acquisition time point (ALSPAC-20 MRI-I and ALSPAC-30 MRI-II) as well as by clinical classification (e.g., PE, control).

ALSPAC, Avon Longitudinal Study of Parents and Children; MRI, magnetic resonance imaging; PE, psychotic experience; PLIKS-18, Psychotic-like Symptoms-18.

The MRI data acquisition for this group was performed at the Cardiff University Brain Imaging Research Centre (CUBRIC) utilizing a 3T GE HDx system (GE Medical Systems) equipped with an 8-channel head coil for signal reception. High-resolution T1-weighted images were obtained through a 3-dimensional (3D) fast spoiled gradient-echo technique, using the following parameters: TR = 7.8 ms, TE = 3.0 ms, flip angle = 20°, and voxel size = 1 mm^3^ isotropic.

#### ALSPAC-30 MRI-II

A second round of data acquisition was carried out between 2019 and 2022. Healthy individuals and participants with PEs who had MRI data acquired at age 20 were invited again for brain imaging and reassessed using the PLIKS interview at age 30. Of the 289 participants who were rescanned, 10 were excluded due to quality issues. In this group, 29 participants were originally identified as suspected (PLIKS-18 = 1), 26 had definite PEs (PLIKS-18 = 2), and 14 were classified as clinical disorder (PLIKS-18 = 3), while 210 participants presented no PEs (PLIKS-18 = 0). All MRI data for this group were acquired at CUBRIC on a 3T Siemens MRI system (Siemens), model Connectom, using a 32-channel head coil. T1-weighted structural images were obtained with a 3D magnetization-prepared rapid acquisition gradient-echo (MPRAGE) sequence (TR = 2.3 ms, TE = 2 ms, inversion time = 0.857 ms, flip angle = 9°, voxel size = 1 mm isotropic).

#### Longitudinal Subsample

A total of 113 participants were scanned at both time points, yielding 226 longitudinal scans to examine brain-age change in PEs. To characterize that change, we applied 2 complementary classifications:

1. Longitudinal PEs 1 (LPEs-1), based on the PLIKS-18 classification, distinguishes participants who were PE negative at baseline from participants who were PLIKS-18 positive.
2. Longitudinal PEs 2 (LPEs-2), a trajectory classification, uses PLIKS scores at both ages, 18 and 30, and splits the cohort into 4 mutually exclusive groups: control (*n* = 56, PLIKS-18 = 0 and PLIKS-30 = 0); remitted (*n* = 35, PLIKS-18 ∈ [1–3]; PLIKS-30 = 0); persistent (*n* = 17, PLIKS-18 ∈ [1–3]; PLIKS-30 ∈ [1–3]); and incident (*n* = 5, PLIKS-18 = 0; PLIKS-30 ∈ [1–3]).

Descriptive statistics for both LPEs-1 and LPEs-2 are presented in Table S1.

### Statistical Analysis

#### Brain-Age Evaluation

**Figure 2:**
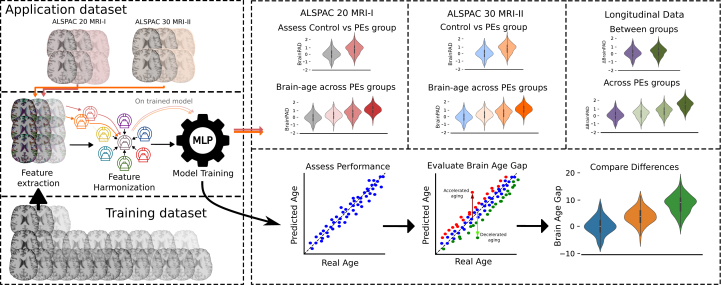
Comprehensive depiction of the methods used to estimate brain age and evaluate the brain-predicted age difference (BrainPAD) across psychotic experience (PE) groups. The diagram outlines the process starting with the training data and ALSPAC (Avon Longitudinal Study of Parents and Children) acquisition sessions. Feature extraction is performed on magnetic resonance imaging (MRI) data from both ALSPAC acquisition time points and training data. A single site reference is created harmonizing the training dataset together. Each acquisition of the application dataset is then harmonized separately to this reference. The model performance is assessed by comparing predicted age against real age. The BrainPAD is evaluated by analyzing the difference between predicted brain age and actual age. This approach is used to assess differences in BrainPAD between the control and PE groups across varying levels of psychotic symptom severity and over a 10-year longitudinal follow-up period. MLP, multilayer perceptron.

Details of the brain age model’s training dataset, harmonization, and training procedure are provided in the Supplement (see Tables S2 and S3). Figure 2 shows the workflow followed. Its performance was evaluated on the test data using 3 key metrics: mean absolute error (MAE), *R*^2^, and Pearson’s *r*. At each time point, the BrainPAD was defined as the difference between actual and predicted age. Group comparability was confirmed prior to modeling. Normality of age distributions was assessed with the Shapiro-Wilk test; equality of variances was assessed with Levene’s test, followed, where appropriate, by an independent-samples *t* test, Welch’s *t* test, or Mann-Whitney *U* test. Sex distributions were compared using χ^2^ tests.

#### Statistical Inference

We analyzed ALSPAC-20/30 BrainPADs with one linear mixed-effects model (LMM). Fixed effects were time (baseline vs. follow-up), PE status at age 18 years (PLIKS-18: 0 = control, 1–3 = suspected, definite, clinical disorder), their interaction (time × PLIKS-18), the Euler number, and sex; the Euler number adjusts for segmentation quality (28, 29, 30). The estimated total intracranial volume was omitted to avoid masking PE-related neurobiology (31,32). A random intercept per participant controlled for repeated measures. Thus, the model formula was:

(1) $${\text{BrainPAD}}_{\mathrm{ij}}^{c}=\beta_{0}+\beta_{1}{\text{Time}}_{ij}+\beta_{P}^{T}P_{i}+\beta_{PT}^{T}\left({\text{Time}}_{ij}P_{i}\right)+\beta_{2}{\text{Sex}}_{i}+\beta_{3}{\text{Euler}}_{ij}+b_{0i}+\varepsilon_{ij}\text{,}\;b_{0i}\;∼\;Ν\left(0,\sigma_{b}^{2}\right)$$

In equation 1, *BrainPAD*^*c*^_*ij*_ represents the age-bias–corrected BrainPAD for participant *i* at visit *j*. *Time*_*ij*_ is a binary indicator of cohort wave, coded 0 for the 20-year scan (ALSPAC-20 MRI-I) and 1 for the 30-year scan (ALSPAC-30 MRI-II). *P*_*i*_ is the 4-element dummy vector that encodes the participant’s PLIKS-18 status. The product *Time*_*ij*_ *× P*_*i*_ supplies the 3 time × PLIKS-18 interaction terms, allowing group differences in the 10-year change. *Sex*_*i*_ is coded 0 for females and 1 for males, and *Euler*_*ij*_ is the Euler number. The random effect (*b*_0*i*_) is the participant-specific intercept. Finally, *ε*_*ij*_ is the residual error term. Together, these components yield a mixed model that adjusts for sex and segmentation quality, accommodates both within-subject correlation and group-specific error variance, and directly tests the cross-sectional, severity, and longitudinal hypotheses of interest.

Post hoc contrasts were obtained using estimated marginal means (EMMs) setting up contrasts that map directly onto our hypotheses.

1. Cross-sectional group gap (hypothesis 1): at each visit (ALSPAC-20 MRI-I and ALSPAC-30 MRI-II), we compared control (PLIKS-18 = 0) with the pooled PLIKS-18-positive categories (PLIKS-18 = 1–3).
2. Severity trend (hypothesis 2): A linear contrast across the 4 PE levels was tested at both visits.
3. Longitudinal divergence (hypothesis 3): We contrasted the change in control participants with that of PLIKS-18-positive participants longitudinally. To explore the longitudinal changes more finely, we reestimated the same LMM after replacing the baseline PLIKS-18 term (LPEs-1) with the PLIKS trajectories along the longitudinal sample explained in Table S1 (LPEs-2) while taking the symptoms’ evolution across time into account.

We grouped the 6 tests into 3 families, applied Benjamini–Hochberg false discovery rate (FDR) control within each (*q* ≤ .050), and calculated Cohen’s *d* from EMM differences over residual variance. Due to the cohort’s narrow age span, we bias corrected brain-age scores before analysis rather than modeling age as an additional covariate. We applied Zhang’s bias correction method (33) after Cole’s post hoc correction (34), without standardization. We fit the predicted-versus-actual age regression on the validation set, thereby preventing leakage.

We repeated the analysis after discarding the worst 5% of scans by standardized Euler number to rule out image-quality artifacts. Furthermore, longitudinal reliability in PE-negative control individuals (LPEs-1, *n* = 61) was gauged with the intraclass correlation coefficient (ICC [3,1]), standard error of measurement (SEM), Spearman’s rank correlation, and Bland-Altman agreement analysis. All statistical analyses were performed using Python 3.11.6 and R 4.4.3 relying on the *emmeans* and *nlme* libraries.

#### Depression-Adjusted Sensitivity Analysis

We reran the mixed model with an age 17 ICD-10 diagnosis of depression as covariate (35). Two study variables were used: FJCI1001 (ICD-10 diagnosis of depression, 1 = yes, 0 = no) and FJCI1002 (subthreshold depressive symptoms, 1 = yes, 0 = no). Summing the binaries created a 0 to 2 scale: 0 = none, 1 = diagnosis or symptoms, 2 = both. This variable was entered as an ordinal fixed effect and interaction term with PLIKS-18 status (LPEs-1) or trajectory (LPEs-2). Observations with missing data on either FJCI variable were excluded.

#### Model-Free Validation Analysis

To address potential machine learning confounds, we added 2 unsupervised analyses: 1) we built a principal component analysis (PCA)– derived age axis from the longitudinal control data, and its associations with PLIKS-18 were tested within each wave; 2) per-participant mean absolute annualized percent change across features, tested versus PLIKS-18. Full details are provided in the Supplement.

## Results

### Harmonization and Model Performance

**Figure 3:**
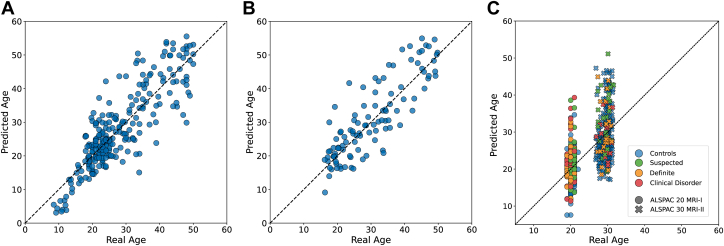
Performance and external validity of the brain-age model. **(A)** Chronological vs. predicted age for the internal holdout test set (*n* = 263). The model achieves a mean absolute error (MAE) of 3.72 years, Pearson *r* = 0.86, and *R*^2^ = 0.73. **(B)** Generalization to the independent AgeRisk cohort (*n* = 105) MAE = 4.27 years, *r* = 0.85, and *R*^2^ = 0.72. **(C)** Brain-age predictions for the ALSPAC (Avon Longitudinal Study of Parents and Children) cohorts (ALSPAC-20 magnetic resonance imaging [MRI] I, circles; ALSPAC-30 MRI-II, crosses), color coded by psychotic-like symptom severity at 18 years.

Harmonization assumptions failed, so we used nonparametric ComBat-GAM, cutting mean residual scanner variance from η^2^ = 0.203 to 0.021 (see Table S4 and Figure S1). The brain-age model demonstrated an MAE (95% CI) of 3.72 (3.31 to 4.14) years, a Pearson *r* (95% CI) of 0.86 (0.82 to 0.89), and an *R*^2^ (95% CI) of 0.73 (0.67 to 0.79) in the internal holdout test. When we similarly applied the model to the AgeRisk dataset (36), the results showed an MAE (95% CI) of 4.27 (3.69 to 4.90) years, an *R*^2^ (95% CI) of 0.72 (0.61 to 0.79), and an *r* (95% CI) of 0.85 (0.79 to 0.90). Performance for the 2 ALSPAC waves is summarized in Table S5. All performance metrics were computed from the raw, bias-uncorrected predictions. For clarity, the main text figures show bias-corrected values only. The uncorrected plots are in Figure S2, the correction’s impact is illustrated in Figure S3, and the corrected performance metrics are listed in Table S6, enabling readers to validate the bias correction step. BrainPAD-age plots for the internal holdout test, external test, and ALSPAC cohorts are shown in Figure 3.

### Statistical Analysis Results

At 20 years, the 3 PE severity groups differed slightly in age (Kruskal-Wallis, *H* = 27.62, *p* < .001), although the largest mean gap was only about 0.3 years, and group variances were equal (Levene’s test, *p* = .160). At the 30-year visit, no age difference was detected (Kruskal-Wallis, *H* = 0.18, *p* = .192; Levene’s test *p* = .753). Sex was balanced at baseline (χ^2^_1_ = 1.42, *p* = .233) but shifted at follow-up (χ^2^_1_ = 8.29, *p* = .004). See the results for all tests in Table 1 and Tables S1 and S7.

Using the LMM formula mentioned in Methods and Materials, our initial random-intercept fit showed heteroscedasticity (Breusch-Pagan, *BP* = 125.94, *p* < .001). We Yeo-Johnson transformed the BrainPAD and let residual variance vary by PLIKS-18 group, but spread still increased with fitted values. Adding an exponential variance function and a random time slope fully stabilized the residuals, which were normal (Shapiro-Wilk, *W* = 1.00, *p* = .954) and homoscedastic (Breusch-Pagan, *BP* = 0.62, *p* = .431). Diagnostic plots can be found in Figure S4. Results for the main hypothesis of the manuscript are shown in Table 2.

**Table 2:** Contrasts From the Mixed Model

Hypothesis	Contrast	Δ, Years	*p*	*q*	Cohen’s *d* [95 % CI]
H1—Cross-Sectional Gap	20 years: control vs. PE	0.32 ± 0.13	.014	.029	0.70 [0.14 to 1.27]
	30 years: control vs. PE	0.22 ± 0.15	.153	.153	0.22 [−0.08 to 0.51]
H2—Severity Trend	20 years: linear contrast	1.34 ± 0.68	.049	.098	1.32 [0.00 to 2.64]
	30 years: linear contrast	−0.40 ± 0.95	.677	.677	−0.39 [−2.25 to 1.47]
H3—Longitudinal Divergence	LPEs-1 (Δcontrol − ΔPE)	−0.10 ± 0.20	.604	.604	−0.23 [−0.79 to 0.33]
	LPEs-2 (Δcontrol − ΔPE)	0.07 ± 0.22	.751	.751	0.10 [−0.36 to 0.51]

The Δ are values power-transformed bias-corrected differences in the brain-predicted age difference (years ± SE). H1 led us to test the cross-sectional gap between the control and the pooled PE groups at each visit; H2 led us to test the linear severity trend within each visit; and H3 is the basis for testing longitudinal divergence as the difference in 10-year slopes.

H, hypothesis; LPE, longitudinal PE; PE, psychotic experience.

The second LMM, which models longitudinal change via LPEs-2 trajectories, was fitted only on the longitudinal subsample (*n* = 113 participants, 226 scans), as trajectory definitions require PLIKS data at both time points. Adding a single-visit class produced rank deficiency, so single-visit cases were excluded. Residuals were normal (*W* = 1.00, *p* = .878) and homoscedastic (Breusch-Pagan, *BP* = 0.05, *p* = .813). All diagnostic plots are provided in Figure S4. Effect sizes for cross-sectional pairwise contrasts can be found in Table S8. Full fixed-effects output, covariates (Tables S9 and S10), and longitudinal pairwise contrasts (Table S11) are provided in the Supplement.

**Figure 4:**
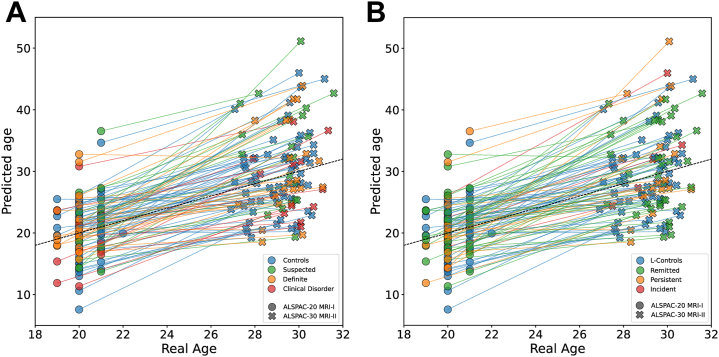
Predicted brain age across the 2 magnetic resonance imaging (MRI) waves in the ALSPAC (Avon Longitudinal Study of Parents and Children) cohort. **(A)** Participants are colored according to their psychotic experience (PE) group at age 18 (longitudinal PEs 1 [LPEs-1]): control, suspected, clinical disorder, and definite. **(B)** The same individuals are recolored by longitudinal symptom course between 18 and 30 years: longitudinal controls and remitted, persistent, and incident cases (LPEs-2). Circles correspond to the first MRI acquisition (ALSPAC-20 MRI-I), and crosses correspond to the second MRI (ALSPAC-30 MRI-II). Each line links the 2 scans of a single participant, illustrating within-subject change in predicted age.

**Figure 5:**
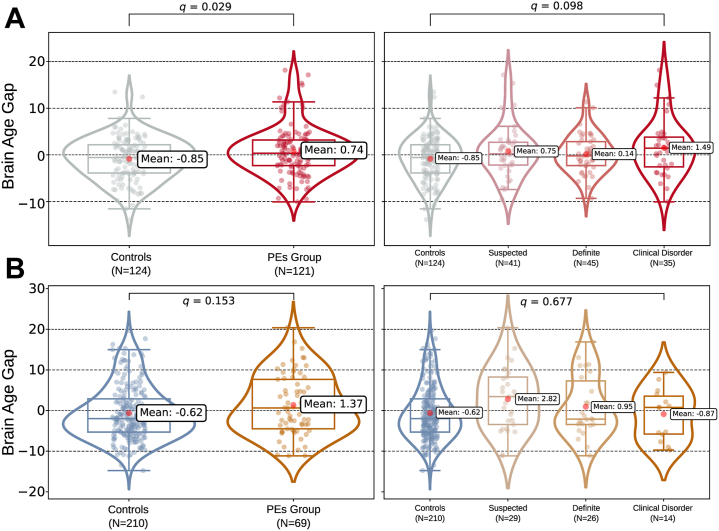
The brain-predicted age difference (BrainPAD) in youths with psychotic experiences (PEs) across 2 waves. Raincloud plots show the mean bias-corrected BrainPAD per group. **(A)** First acquisition (ALSPAC [Avon Longitudinal Study of Parents and Children] 20 magnetic resonance imaging [MRI] I) (top): The mean BrainPAD was higher in the combined PE group than in the control group. Within PEs, the BrainPAD increased linearly with symptom severity. **(B)** Follow-up (ALSPAC-30, MRI-II) (bottom). The PE group exceeded the control group again, but not significantly so, and the within-PEs linear trend did not reach significance either.

**Figure 6:**
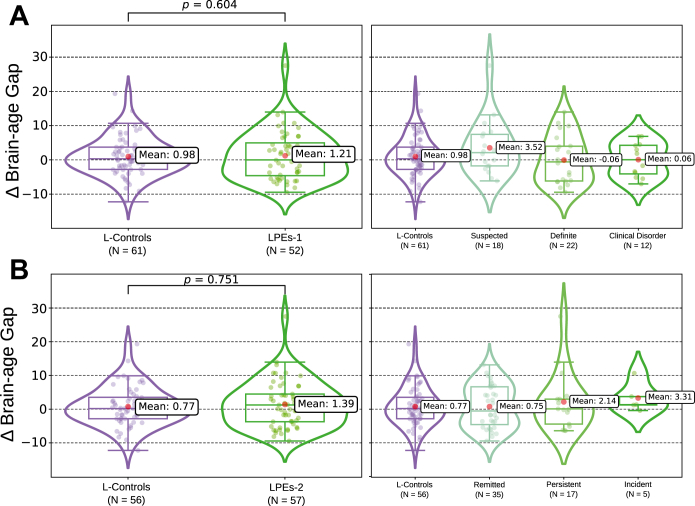
Longitudinal differences in the brain-predicted age difference (Δbrain-age gap) across psychotic experience (PE) groups. **(A)** Longitudinal PEs 1 (LPEs-1) definition. Left: longitudinal control participants (L-controls) vs. participants who reported any PEs using the Psychotic-like Symptoms (PLIKS)-18 definition. Right: the LPEs-1 cohort stratified by clinical severity into suspected, definite, and clinical disorder subgroups. **(B)** LPEs-2 definition. Left: L-controls vs. participants with PEs with the LPEs-2 definition. Right: LPEs-2 subdivided by symptom course into remitted (PEs resolved), persistent (PEs present at both waves), and incident (new-onset PEs) groups.

At age 20, participants with PEs showed a mean BrainPAD larger than that of control participants (Cohen’s *d* = 0.70; 95% CI, 0.14 to 1.27; *p* = .014, *q* = .029). A linear trend across PLIKS-18 severity levels was also significant before FDR correction (Δ = 1.34 ± 0.68 years per level; *p* = .049, *q* = .098). By age 30, the control-PE difference was 0.22 ± 0.15 years and nonsignificant (*p* = .153). Longitudinal contrasts of 10-year change, represented in Figure 4, Figure 5, Figure 6, were also nonsignificant (all *p*s ≥ .600).

### Sensitivity Analysis: Euler Number

After excluding the lowest 5% of scans ranked by Euler number (*n* = 499/524), the primary 20-year control-PE effect remained virtually unchanged (*d* = 0.66, *q* = .048; Δ*d* = −0.04). The dose-response across PLIKS levels at 20 years stayed positive but was no longer nominally significant. All other contrasts, including the 30-year wave, were unaffected (see Tables S12 and S13 for full statistics).

### Sensitivity Analysis: Depression

Restricting the sample to participants with complete ICD-10 depression data (484 scans) and adding depression (main effect + PE × depression interaction) barely changed the model fit (ΔAkaike’s information criterion = +5.9) and left the primary PE effects intact. Depression showed a small, exploratory association with BrainPAD (*d* = 0.23; 95% CI, 0.03 to 0.43; *p* = .027, uncorrected). At 20 years, the PE group still appeared older than the control group (*d* = 0.69, *q* = .022); at 30 years, the contrast remained nonsignificant. Full statistics and residual diagnostics are provided in Tables S14 to S16.

### Longitudinal Reliability of Brain-Age Estimates

In control participants who were rescanned (LPEs-1, *n* = 61), bias-corrected BrainPAD drifted minimally across 10 years (Δ = 0.01 years; limits ±6 years) (Figure S5). Agreement was modest (ICC [3,1] [95% CI] = 0.42 [0.19 to 0.60]; SEM = 3.11 years; Spearman ρ = 0.41). BrainPAD change was virtually independent of chronological aging (*r* = 0.04), and mixed-effects modeling confirmed that the predicted corrected age tracked chronological age with a slope of 1.01 ± 0.08 with a near-zero intercept.

### Model-Free Validation

Along the control-trained PCA age axis, PLIKS showed small positive trends at both waves, clearest after ComBat-GAM (MRI-I Spearman’s ρ = 0.138, *p* = .030; MRI-II ρ = 0.147, *p* = .014). By contrast the mean absolute annualized percent change showed no association with PLIKS (unharmonized: Spearman’s ρ = −0.097, *p* = .306; harmonized: ρ = −0.095, *p* = .317) (see Table S17 and Figure S6).

## Discussion

In this study, we investigated how PEs in nonclinical individuals relate to brain age at ages 20 and 30. Results show that participants with PEs reported at 18 predicted a significantly larger BrainPAD at 20 than in control participants, suggesting underlying structural brain alterations. Further analysis demonstrated that the magnitude of this increase correlated positively with the severity of PEs reported at age 18, with individuals reporting more severe PEs showing a more pronounced difference (*d* = 1.32; 95% CI, 0.00 to 2.64; uncorrected *p* = .049), although this trend did not remain significant after FDR correction (*q* = .098).

By the 30-year visit, the BrainPAD no longer differed between the PE and control groups (*q* = .153), and none of the 10-year change contrasts were significant. With the LPEs-1 definition, higher severity groups showed nonsignificant point estimates, indicating smaller 10-year changes in the BrainPAD than the control group (ΔDefinite *d* = 0.06; 95% CI, −0.44 to 0.57; ΔClinical *d* = 0.47; 95% CI, −0.25 to 1.20) (Table S8). Furthermore, with the LPEs-2, remitted (*d* = 0.06; 95% CI, −0.76 to 0.88) and persistent (*d* = −0.08; 95% CI, −1.13 to 0.98) groups showed little change versus the control group, whereas incident cases shifted more (*d* = −0.29; 95% CI, −2.06 to 1.49) (Table S11). Overall, the 20-year BrainPAD narrows, not widens, over the next decade, suggesting possible normalization, but wide confidence intervals leave the mechanism uncertain. This finding may reflect limited power, because only 113 participants contributed scans at both waves, and the highest severity cases were underrepresented at follow-up. Power simulations (α = 0.05, 1000 draws) (Table S18) showed high power for the observed cross-sectional effects (≥0.90) but very low power for longitudinal contrasts (∼0.05), so 10-year changes should be interpreted with caution.

Our finding at age 20, absent at age 30, matches 2 recent studies. First, Hua *et al.* (37) showed youths at future risk for psychosis already had bigger gaps, so the gap predates onset. Second, our fading gap mirrors the meta-review by Merritt *et al.* (38), in which high-risk individuals exhibited early region-specific gray matter loss that appeared to normalize when symptoms remitted. Although our 10-year change estimates were imprecise, our results similarly suggest that the PE-control gap closes. A potential explanation for our findings is to view the BrainPAD as a composite readout of atypical maturation. Adolescence and the early 20s are dominated by pruning and myelination; if that process runs too quickly or intensely, a leading theoretical perspective on the pathogenesis of schizophrenia (39, 40, 41, 42), the cortex can look older than expected. This explanation fits our age 20 result. It also accommodates the possible loss of the gap by age 30; normal thinning in control participants can erase the gap without neurodegeneration.

Removing 5% of scans with the lowest Euler numbers (retaining 499/524 images) slightly decreased the control-PE gap at age 20 from 0.32 ± 0.13 to 0.30 ± 0.13 (*d* = 0.66, *q* = .048). The linear trend across PLIKS levels at the same age also inched downward (β = 1.27 ± 0.70; Δβ = −0.07 vs. the unfiltered model). These results show that Euler number screening curbed segmentation errors, supporting BrainPAD as a genuine effect. Adding depression as a covariate from the ICD-10 results and its interactions with PEs in the inference models only had a modest main effect (*d* = 0.23; 95% CI, 0.03 to 0.43; *p* = .027, uncorrected) and did not alter the key PE contrasts: The baseline PE group still showed a significant and moderately higher BrainPAD (*d* = 0.69; 95% CI, 0.16 to 1.23; *q* = .022), but the difference at follow-up remained negligible. Thus, the elevated BrainPAD seems specific to PEs, not depression. Model-free validations echoed the main result: Small offset at the first acquisition on a control-trained PCA age axis, with annual percentage change being unrelated to PLIKS.

Our model uses a transparent, feature-based design (43,44) and still scores well out of sample (MAE = 4.27 years, *R*^2^ = 0.75, *r* = 0.82) with little loss after harmonization. On ALSPAC-20 data, MRI-I performs slightly better, because the cohort matches the training set’s core age range. Deep learning models can reduce error further but sacrifice interpretability, a drawback for clinical use (45, 46, 47). Among the 61 healthy control participants rescanned a decade apart (LPEs-1), the bias-corrected BrainPAD was stable: The mean drift was very small (Δ = 0.01 year; limits of agreement = ±6 years). However, the ICC was modest (ICC [3,1] = 0.42, [0.19 to 0.60]); since the age range is narrow, between-subjects variance is low, and thus even slight noise lowers the ICC. To remove systematic age bias, we applied the Zhang method (33) after Cole’s post hoc correction (34), estimating the adjustment parameters in our validation partition and thereby avoiding any data leakage. Other strategies can inflate accuracy (48,49), but this approach avoids it.

A further caveat concerns FastSurfer’s CNN, which replicates FreeSurfer outputs from the Desikan-Killiany-Tourville atlas, built on data from 19- to 33-year-old individuals (50). ALSPAC’s 20-year scans sit at the range’s lower edge, so template mismatch may add minor age-related surface and parcellation errors. Because this bias should be uniform within each age band, the direction of the PE-control difference is probably sound, although its exact magnitude may be difficult to measure. This limitation weighs heavier longitudinally: As participants age, atlas mismatch shrinks, so decade-long brain-age shifts may stem from better algorithmic fit, not biology. In our cohort, the interaction between PE symptoms and time was nonsignificant and underpowered; therefore, atlas-related drift might have obscured a modest effect. Confirming atypical brain-age trajectories will require age-specific segmentation, refined longitudinal pipelines, and larger cohorts.

The findings from recent research on white matter alterations in individuals at ultra-high-risk of transitioning to psychosis (51) suggest that a DTI-based brain-age approach could provide additional valuable insights into brain development in individuals with PEs. Furthermore, other brain-age studies suggest that functional connectivity brain-age scores are linked to genetic variations associated with schizophrenia subtypes (52). Multimodal brain-age models may predict psychosis progression better, thereby enhancing early intervention and biomarker value. Finally, adding an early adolescent follow-up would reveal whether the BrainPAD predates or emerges during late adolescence (52, 53, 54).

Although we trained the brain-age model on a heterogeneous multisite dataset and confirmed its external validity on the AgeRisk sample, the ALSPAC-PE subsample remains demographically and geographically circumscribed. Population differences in environment, culture, and genes may shift PE prevalence and neurodevelopment, thereby changing the BrainPAD size or direction. Finally, despite harmonization, the generalizability of the absolute BrainPAD to scanners with markedly different field strengths, pulse sequences, or vendor-specific gradient nonlinearities has not been tested. Replication in multiethnic population cohorts, and across a wider range of imaging platforms, will be essential to establish the generalizability of the current results to other settings.

### Conclusions

PEs reported at 18 predicted an increased BrainPAD at 20, but the difference disappeared by 30. Although gap size roughly tracked symptom severity, the trend fell below the cutoff for FDR-corrected significance. These findings raise key questions: Do PEs accelerate brain maturation, do preexisting maturational deviations predispose individuals to PEs, or is the relationship bidirectional? Disentangling these possibilities will require larger, multiwave cohorts that start before adolescence and extend into midadulthood, ideally enriched with diffusion and functional connectivity data. Understanding these mechanisms is key to biomarker development and better prevention for psychosis-risk groups.

## Supplementary Material

Supplemental Methods, Results, Figures S1–S6, and Tables S1–S18

Key Resources Table
