# Supplementary material for: Increased Brain-Age Gap in Young Adults With Psychotic Experiences
Source: Biol Psychiatry Glob Open Sci. 2025 Oct 30;6(2):100643. doi: 10.1016/j.bpsgos.2025.100643 (PMC13007526; doi:10.1016/j.bpsgos.2025.100643)
Supplement: Supplemental Methods, Results, Figures S1–S6, and Tables S1–S18 [file mmc1.pdf]

SUPPLEMENTARY INFORMATION

Increased Brain-Age Gap in Young Adults With Psychotic Experiences

Navarro-González *et al.*

SUPPLEMENTARY MATERIALS

ALSPAC Study population

The ALSPAC study originally invited pregnant women resident in Avon, UK with expected dates of delivery between 1st April 1991 and 31st December 1992. 20,248 pregnancies have been identified as being eligible and the initial number of pregnancies enrolled was 14,541. Of the initial pregnancies, there was a total of 14,676 foetuses, resulting in 14,062 live births and 13,988 children who were alive at 1 year of age. When the oldest children were approximately 7 years of age, an attempt was made to bolster the initial sample with eligible cases who had failed to join the study originally. As a result, when considering variables collected from the age of seven onwards (and potentially abstracted from obstetric notes) there are data available for more than the 14,541 pregnancies mentioned above: The number of new pregnancies not in the initial sample (known as Phase I enrolment) that are currently represented in the released data and reflecting enrolment status at the age of 24 is 906, resulting in an additional 913 children being enrolled (456, 262 and 195 recruited during Phases II, III and IV respectively). The phases of enrolment are described in more detail in the cohort profile paper and its update (1–3). The total sample size for analyses using any data collected after the age of seven is therefore 15,447 pregnancies, resulting in 15,658 foetuses. Of these 14,901 children were alive at 1 year of age.

Of the original 14,541 initial pregnancies, 338 were from a woman who had already enrolled with a previous pregnancy, meaning 14,203 unique mothers were initially enrolled in the study. As a result of the additional phases of recruitment, a further 630 women who did not enrol originally have provided data since their child was 7 years of age. This provides a total of 14,833 unique women (G0 mothers) enrolled in ALSPAC as of September 2021.

G0 partners were invited to complete questionnaires by the mothers at the start of the study and they were not formally enrolled at that time. 12,113 G0 partners have been in contact with the study by providing data and/or formally enrolling when this started in 2010. 3,807 G0 partners are currently enrolled.

The PLIKSi semi-structured interview begins with a short set of sleep-related questions, nightmares, night terrors and sleep-walking, to ease children into discussing unusual experiences, and then poses 12 core items that tap the three principal types of positive psychotic symptoms: hallucinations (visual and auditory), delusions (being watched, persecuted, having thoughts read, ideas of reference or control, grandiose beliefs and other unspecified delusions) and bizarre experiences (thought broadcasting, insertion and withdrawal). Seven of these core questions

come from the Diagnostic Interview Schedule for Children-IV (DISC-IV) and five from sections 17–19 of the Schedules for Clinical Assessment in Neuropsychiatry (SCAN) version 2.0 (4), all slightly modified after pilot testing, with responses coded strictly according to SCAN glossary definitions and rating rules (5). Psychotic experiences (PEs) were classified based on the Schedule for Clinical Assessment in Neuropsychiatry (SCAN) (4).

Prior work has examined whether particular PEs carry different weight in predicting outcomes. In the ALSPAC cohort, (6) analyzed participants at age 18, and (7) extended this to age 24. Both studies found that the number of PEs phenomena and, to a lesser extent, their persistence, matters more than which specific symptom occurs. In our sample, most individuals who met PEs criteria reported just one experience (62.6 %); two experiences were present in 21 %, and three in 8.8 %. Auditory hallucinations were most common, followed by visual hallucinations and feelings of being spied upon. Because individual symptoms were relatively rare and prior evidence shows overall load is what predicts outcomes, the present study was under-powered, and not strongly motivated, to test each symptom separately.

Dataset		N	Age (mean years)	Age SD	Age range	Sex (M/F)	
Longitudinal Controls		61	20.21 / 29.08	0.61 / 1.11	19-22 / 27-31	21/40	Mann-Whitney *U* $P = .494$ Sex X^2 $P = 1.000$
Longitudinal PEs		52	20.12 / 29.54	0.65 / 1.02	19-21 / 27-32	20/37	
LPEs-1	*Suspected (PLIKS-18 = 1)*	18	20.56 / 29.40	0.51 / 1.17	20-21 / 27-31	8/10	
	Definite (PLIKS-18 = 2)	22	19.77 / 29.51	0.43 / 0.91	19-20 / 27-31	7/15	
	Clinical Disorder (PLIKS-18 = 3)	12	20.08 / 29.83	0.79 / 1.01	19-21 / 28-31	3/9	
Longitudinal Controls		56	20.21 / 29.97	0.62 / 1.11	19-22 / 27-31	19/37	Mann-Whitney *U* $P = .514$ Sex X^2 $P =.000$
Longitudinal PEs		57	20.12 / 29.52	0.63 / 1.03	19-21 / 27-32	20/37	
LPEs-2	*Remitted PLIKS-18 ∈ [1-3], PLIKS-30 = 0*	35	20.17 / 29.59	0.71 / 1.10	19-21 / 27-32	13/22	
	Persistent PLIKS-18 & PLIKS-30 ∈ [1-3]	17	20.00 / 29.46	0.50 / 0.87	20-21 / 28-31	5/12	
	Incident PLIKS-18 = 0, PLIKS-30 ∈ [1-3]	5	20.20 / 29.23	0.45 / 1.22	20-21 / 28-31	2/3	
Longitudinal Data		**113**	**20.17 / 29.29**	**0.63 /1.09**	**19-22 / 27-32**	**39/74**	--

Table S1. Demographic profile of the 113 subject longitudinal cohort and its sub-groups. For the data set we list the number of individuals (N), the mean age (baseline / follow-up years) its standard deviation (SD), range, and the male-to-female count. The top half of the table shows (Longitudinal Psychotic experiences 1, LPEs-1) subgroups defined by PLIKS-18 psychotic-experience categories. The lower half shows trajectory-based subgroups derived from PLIKS-18 and PLIKS-30 assessments (Longitudinal psychotic experiences 2, LPEs-2).

Brain-Age Model

Developing a reliable brain-age model involves several key steps. First, a large, diverse dataset linking neuroimaging data with chronological age is essential to ensure accuracy and generalizability. Depending on the approach, brain-age models can either rely on traditional machine learning methods, which often require feature extraction from the brain scans (e.g., gray matter volume or cortical thickness), or employ more advanced techniques like deep learning, where the algorithm automatically learns complex patterns directly from the imaging data without manual feature selection. The chosen algorithm is trained using techniques like regularization, data augmentation, and cross-validation to improve performance and reduce overfitting. In our study, we use a brain-age model implemented as a multilayer perceptron (MLP), trained on MRI-derived features. Finally, the model is evaluated using metrics such as mean absolute error (MAE), Pearson correlation with chronological age (r), or the coefficient of determination (R^2) to assess its accuracy.

Training dataset

To create and evaluate our brain-age prediction model, we compiled a comprehensive dataset consisting of structural T1w MRI scans from a variety of publicly available studies and databases containing only healthy individuals. These include: the Dallas Lifespan Brain Study (DLBS) dataset (8), the Consortium for Reliability and Reproducibility (CoRR) dataset (9), the Neurocognitive Aging Data Release (NeuroCog) (10), the OASIS-1 Dataset (11), the Southwest University Adult Lifespan Dataset (SALD) (12), the Information eXtraction from Images (IXI) dataset (13), the CamCAN repository (available at http://www.mrc-cbu.cam.ac.uk/datasets/camcan/) (14,15), the National Institute of Mental Health Research Volunteer (NIMH-RV) dataset (16), the Movement-related artefacts (MR-ART) dataset (17), the Nathan Kline Institute - Rockland Sample (NKI-RS) dataset (18) and the Welsh Advanced Neuroimaging Database (WAND) (19). Additionally, we included the AgeRisk dataset (20) for external validation of the model. Propensity score matching (21), was used to balance the sample for age and sex. To mirror the age profile of the application cohort (19–32 years), we excluded participants aged 50 years or older from the training set. In total, the dataset provides a diverse and extensive sample for model training, promoting robust generalization across different populations. Table S2 and Figure 1, in the main text, provide additional details regarding the dataset.

Dataset	n	Age (years, mean)	Age σ	Age range	Sex (M/F)	Scanner B0
CamCan	286	36.24	8.62	18 – 50	135 / 151	3.0 T
CoRR	1010	20.80	6.61	6 – 49	527 / 483	3.0 T
DLBS	115	34.35	8.50	20 – 49	49 / 66	3.0 T
IXI	128	33.65	7.76	20 – 49	74 / 54	3.0 T
MR-ART	110	26.62	6.64	18 – 45	47 / 63	3.0 T
NIMH-RV	128	30.09	7.96	20 – 49	45 / 83	3.0 T
NKI-RS	292	32.14	9.36	19 – 50	138 / 154	3.0 T
NeuroCog	172	22.76	3.25	18 – 34	78 / 94	3.0 T
OASIS-1	127	28.10	9.35	18 – 50	83 / 44	1.5 T
SALD	145	31.94	8.72	19 – 50	97 / 48	3.0 T
WAND	115	28.10	8.05	18 – 48	52 / 63	3.0 T
AgeRisk	105	30.30	10.01	16-49	49/56	3.0 T
Training Dataset	**2628**	**27.07**	**9.59**	**6-50**	**1325/1303**	**3.0T / 1.5T**

Table S2. Datasets used for model training and evaluation. All datasets are available online. The row 'whole dataset' sums up all the data used for training. The AgeRisk dataset was used to evaluate the external generalizability of the model. Datasets have been acquired from different scanners and vendors, with different B0 and acquisition parameters.

Segmentation and Feature extraction

From the T1w images, FastSurfer 2.2.0 (22) was employed to extract a total of 653 brain-related quantitative morphological features. FastSurfer uses deep learning to perform brain segmentation based on the Desikan-Killiany-Tourneville atlas (23). Raw native 3D-T1w images were fed into the network for segmentation. FastSurfer conforms each native 3D T1-weighted volume to 1 mm isotropic RAS orientation, rescales intensities to the range 0-255 and then feeds this conformed image, into three neural network models that segment the brain in axial, coronal and sagittal views. Their 2.5D outputs are subsequently fused into a single label map. Immediately after segmentation, FastSurfer, applies an N4 bias-field correction to the conformed image. This corrected volume is not used for inference but supports downstream steps such as estimated total intracranial volume (eTIV) estimation. The pipeline then runs an accelerated surface reconstruction version of FreeSurfer's to generate cortical meshes, surfaces, and morphometric maps. From the combined segmentation and surface outputs we derived the set of quantitative morphological features. Apart from the built-in FastSurfer steps, no additional preprocessing was applied.

FastSurfer is trained on 140 representative subjects from different public cohorts, (ABIDE-II, ADNI, LA5c, and OASIS-1, OASIS-2) balanced with regard to gender, age, and diagnosis. Training age range spanned from 18 to

90 years old. On a subset (N = 20) of the manually labelled Mindboggle-101 benchmark (24), FastSurfer reproduced expert anatomy with a Dice similarity coefficient of 80.19 and 80.65 and an average Hausdorff distance of 0.29 and 0.40 on the subcortical and cortical structures, respectively (22). Complementary paediatric evidence shows that FastSurfer's output closely tracks the volumetric results of FreeSurfer and previously published growth charts, in 448 children aged 4–18 years, yielding a mean Dice of 0.90, 95 % CI [0.79, 0.95] and ICC = 0.87, 95 % CI [0.52, 0.94] across 15 macro-ROIs (25). Because our ALSPAC participants are 19–32 years old, they fall within the validated age range, and comparable segmentation accuracy is anticipated. All FastSurfer outputs were visually inspected and reran any scans with obvious surface errors. If errors persisted the image was discarded.

Up to 31 cortical Regions of Interest (ROIs) were extracted per hemisphere during segmentation. For each cortical ROI, 9 distinct features were measured: average cortical thickness, standard deviation of cortical thickness, gray matter volume, surface area, mean curvature, Gaussian curvature, curvature index, folding index, and the underlying white matter volume. In addition, 29 ROIs were identified in the cerebellum, including 10 lobules per hemisphere, 5 vermis regions, and 4 regions corresponding to the cerebellar cortex and cerebellar white matter. For each cerebellar ROI, the volume was measured. Furthermore, 8 subcortical ROIs were extracted from each hemisphere, with volume calculated for each region. We also measured volumes for 5 ventricular structures and divided the corpus callosum into 5 regions, each with a volume measurement. Additionally, we extracted volumes for the brainstem and optic chiasm. Lastly, 21 whole brain volumes and ratios were calculated, providing a comprehensive overview of global brain measurements. Table S3 show in detail the features extracted.

Harmonization

MRI features were harmonized with ComBat-GAM (26). Sex, age and eTIV were entered as covariates so that their biological effects were preserved. Prior to harmonization we discarded 32 FastSurfer derived variables that either (i) index segmentation quality, (ii) represent lesion or vessel masks, (iii) correspond to very small structures with low test–retest reliability, or (iv) duplicate global volume measures already accounted for by eTIV. This filtering ensured that ComBat-GAM was applied only to anatomically meaningful, well-behaved features.

ComBat-GAM assumes scanner shifts (γ) are approximately normal and scale factors (δ) follow an inverse-Gamma distribution, scanner residuals were screened with pre-defined criteria (|skewness| < 2, |kurtosis| < 7 for $\hat{\gamma}$; $2 \leq a \leq 10$ for $\hat{\delta}$). To harmonize the training and application datasets, first, every scan in the training set was harmonized together to establish a single, site reference scale. Each test wave was then processed in isolation to avoid data-leakage: for ALSPAC-20 MRI-I the ComBat-GAM parameters were learned from the ALSPAC-20

MRI-I control subjects only and applied to the corresponding PLIKS participants; the same control-anchored procedure was repeated for ALSPAC-30 MRI-II. Thus, both test waves were mapped onto the reference scale defined by the training data without letting information from the test sets influence that initial harmonization.

Machine learning model

The training dataset was randomly split into a 60%/30%/10% ratio for training, validation, and a hold-out test set. For each feature, values below the 2.5th or above the 97.5th percentile (estimated on the training split) were clipped and all features were min-max scaled to (-1, 1). The resulting 621 harmonized MRI features constituted the input vector.

We then trained a multilayer perceptron (MLP) with one hidden layer with 16 units (batch normalization, ReLU activation and 35 % dropout) and a linear read-out whose sigmoid output was rescaled to years. Optimisation used the Smooth-L1 loss (Huber, $\beta = 3$) and Adam optimizer (learning rate = 2×10^{-4}), with L2 weight-decay (10^{-3}) to limit weight magnitude and an additional L1 penalty ($\lambda = 10^{-3}$) to encourage implicit feature selection. Training lasted to up to 100 epochs, invoking early stopping after 10 epochs without validation improvement. For Implementation scikit-learn 0.24 (27), and PyTorch 2.3 (28) were used.

Region	Number of ROIs	Extracted Features
Cortical ROIs	31 x 2 hemispheres x 9 features	Average cortical thickness, standard deviation of cortical thickness, gray matter volume, surface area, mean curvature, Gaussian curvature, curvature index, folding index, underlying white matter volume
Cerebellar ROIs	30	Volume of 10 lobules per hemisphere, 6 vermis regions, and the cerebellar cortex volume and cerebellar white matter volume per hemisphere
Subcortical ROIs	8 x 2 hemispheres	Volume of Thalamus, Caudate, Putamen, Pallidum, Accumbens, Hippocampus, Ventral Diencephalon, Amygdala
Ventricular Structures	13	Volume of 3rd-Ventricle, 4th-Ventricle, 5th-Ventricle, Left Lateral Ventricle, Right Lateral Ventricle, Left Inferior Lateral Ventricle, Right Inferior Lateral Ventricle, choroid plexus (right and left), vessels (right and left), Volume CSF, Volume Ventricle Choroid
Corpus Callosum	5	Volume of Anterior, Central, Mid-Anterior, Mid-Posterior, Posterior Corpus Callosum regions
Brainstem and Optic Chiasm	2	Volume of the brainstem and optic chiasm
Whole Brain Measurements	29	BrainSegVol, BrainSegVol-to-eTIV, BrainSegVolNotVent, BrainSegVolNotVentSurf, CortexVol, CerebralWhiteMatterVol, rhCerebralWhiteMatterVol, lhCerebralWhiteMatterVol, TotalGrayVol, eTIV, SubCortGrayVol, SupraTentorialVol, SupraTentorialVolNotVent, SupraTentorialVolNotVentVox, lhCortexVol, rhCortexVol, SurfaceHoles, lhSurfaceHoles, rhSurfaceHoles, MaskVol, MaskVol-to-eTIV, non-WM-hypointensities, WM-hypointensities, right non-WM-hypointensities, left non-WM-hypointensities, right WM-hypointensities, left WM-hypointensities, right unsegmented WM, left unsegmented WM.
**Avoided features before harmonization**	VentricleChoroidVol, Volume_mm3_Left-choroid-plexus, Volume_mm3_Right-choroid-plexus, Volume_mm3_WM-hypointensities, Volume_mm3_Right-WM-hypointensities, Volume_mm3_Left-WM-hypointensities, Volume_mm3_non-WM-hypointensities, Volume_mm3_Right-non-WM-hypointensities, Volume_mm3_Left-non-WM-hypointensities, Volume_mm3_Left-vessel, Volume_mm3_Right-vessel, Volume_mm3_Optic-Chiasm, Volume_mm3_5th-Ventricle, lhSurfaceHoles, rhSurfaceHoles, SurfaceHoles, MaskVol, MaskVol-to-eTIV, BrainSegVol, BrainSegVolNotVent, BrainSegVolNotVentSurf,BrainSegVol-to-eTIV, SupraTentorialVol, SupraTentorialVolNotVent, SupraTentorialVolNotVentVox, CortexVol, TotalGrayVol, CerebralWhiteMatterVol, lhCerebralWhiteMatterVol, rhCerebralWhiteMatterVol, SubCortGrayVol, eTIV	

Table S3. Features extracted and MLP parameters used during the training pipeline.

Harmonization assumptions

scanner name	count	|skew|	|kurt|	$\hat{\gamma}$ status	a	$\hat{\delta}$ status
CARDIFF_MPRAGE	115	0.19	0.29	OK	15.93	flag
CamCan_SIEMENS_MAGNETOM_TrioTim_syngo_MR_3T	286	0.02	0.61	OK	18.98	flag
CoRR_BMB_1_Siemens_TrioTim	43	1.05	2.67	OK	6.86	OK
CoRR_BNU_1_Siemens_TrioTim	57	0.20	0.35	OK	11.03	flag
CoRR_BNU_2_Siemens_TrioTim	56	0.14	0.43	OK	8.27	OK
CoRR_HNU_1_GE_Discovery MR750	20	0.43	4.57	OK	5.67	OK
CoRR_IACAS_GE_Signa HDx	28	0.86	5.49	OK	5.03	OK
CoRR_IBATRT_Siemens_TrioTim	36	0.33	0.31	OK	9.29	OK
CoRR_IPCAS_1_Siemens_TrioTim	30	0.06	0.19	OK	6.91	OK
CoRR_IPCAS_3_Siemens_TrioTim	35	0.04	0.18	OK	5.91	OK
CoRR_IPCAS_4_GE_Discovery	20	0.87	3.34	OK	2.69	OK
CoRR_IPCAS_5_Siemens_TrioTim	22	0.06	0.34	OK	2.11	OK
CoRR_IPCAS_7_Siemens_TrioTim	45	0.39	0.56	OK	6.98	OK
CoRR_NYU_1_Siemens_Allegra	24	0.03	1.74	OK	5.64	OK
CoRR_NYU_2_Siemens_Allegra	169	0.41	2.58	OK	8.58	OK
CoRR_SWU_1_Siemens_TrioTim	20	0.10	1.22	OK	5.50	OK
CoRR_SWU_2_Siemens_TrioTim	27	0.18	0.64	OK	6.73	OK
CoRR_SWU_3_Siemens_TrioTim	23	0.09	0.17	OK	5.91	OK
CoRR_SWU_4_Siemens_TrioTim	234	0.11	0.32	OK	19.80	flag
CoRR_UPSM_1_Siemens_TrioTim	96	0.33	0.65	OK	10.45	flag
CoRR_UWM_GE_Discovery	25	0.67	2.00	OK	6.43	OK
DLBS_SIEMENS_MAGNETOM_TrioTim_syngo_MR_3T	115	0.51	1.16	OK	13.04	flag
IXI_GE_1.5T_system	42	1.12	1.26	OK	4.467	OK
IXI_Philips_Medical_Systems_Intera_3T	86	1.64	3.01	OK	7.77	OK
MR_ART_Siemens_Magnetom_Prisma_3T	110	0.40	0.10	OK	10.07	flag
NIHMFSPGR_Philips_Achieva_3T	51	1.50	4.45	OK	10.18	flag
NIHMMPRAGE_Philips_Achieva_3T	77	0.65	0.70	OK	8.37	OK
NKI_SIEMENS_MAGNETOM_TrioTim_syngo_MR_3T	292	0.01	0.13	OK	15.29	flag
NeuroCog_3T_GE_Discovery_MR750	146	0.40	0.49	OK	13.56	flag
NeuroCog_3T_Siemens_TimTrio_MRI	26	0.05	0.33	OK	6.54	OK
OASIS_1.5-T_Vision_scanner	127	0.89	4.07	OK	9.66	OK
SALD_SIEMENS_MAGNETOM_TrioTim_syngo_MR_3T	145	0.15	0.62	OK	13.70	flag
AgeRisk	105			OK		OK
Harmonized Data	2628	-	-		-	Reference
ALSPAC-20 MRI-I	121			OK		OK
Harmonized Data	2628	-	-		-	Reference
ALSPAC 30 MRI-II	210			OK		OK
Harmonized Data	2628	-	-		-	Reference

Table S4. Batch-level diagnostics for the ComBat-GAM priors. For every scanner we report the sample size and two goodness-of-fit tests used to verify ComBat-GAM's empirical-Bayes assumptions. (i) Additive effects ($\hat{\gamma}$): the $\hat{\gamma}$ estimates across regions should be normally distributed; a batch is flagged when the absolute sample skewness exceeds 2 or the absolute kurtosis exceeds 7. (ii) Multiplicative site effects ($\hat{\delta}$): The $\hat{\delta}$ estimates are expected to follow an inverse-gamma distribution. We therefore, fit an inverse-gamma with maximum-likelihood shape a. A batch is accepted when the value of a is between 2 and 10.

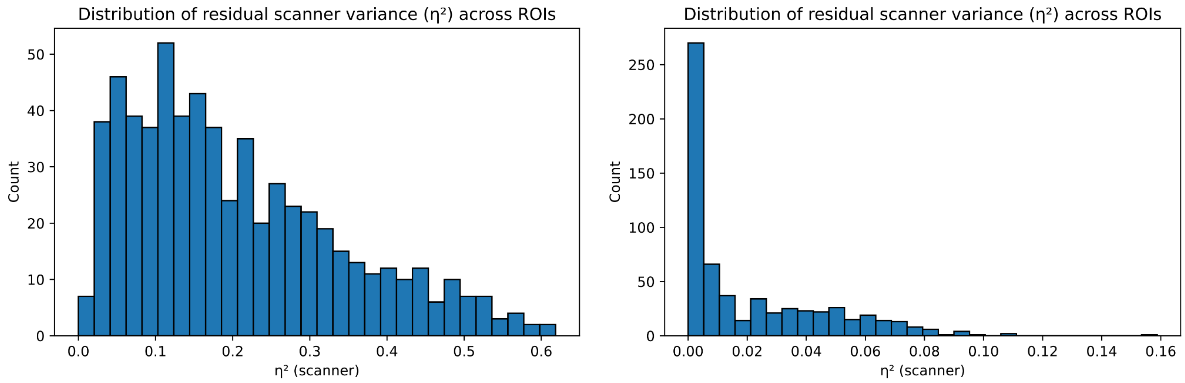

Figure S1. Scanner-related variance (η^2) across brain regions before and after harmonization. For each ROI, a linear mixed-effects model (*roi_value* ~ Age + sex + (1 | Scanner)) was fitted and the proportion of residual variance attributable to the scanner was computed as η^2_scanner = σ^2_scanner / (σ^2_scanner + σ^2_resid). Histograms depict the distribution of η^2_scanner values before (left) and after (right) ComBat-GAM harmonization. the residual scanner variance averaged across features dropped from $\eta^2 = 0.203$ (20.3 % pre-harmonization) to $\eta^2 = 0.021$ (2.1 % post-harmonization)

PLIKS-18	MAE	r Age-pAge	R^2	r BrainPAD-Age	PLIKS-18	MAE	r Age-pAge	R^2	r BrainPAD-Age
**ALSPAC 20 MRI-I**					**ALSPAC 30 MRI-II**				
Controls	3.09 [2.73, 3.49]	0.05 [-0.12, 0.21]	-35.94 [-52.15, -24.13]	-0.14 [-0.31, 0.04]	Controls	4.13 [3.76, 4.51]	0.20 [0.06, 0.33]	-17.85 [-22.59, -13.84]	-0.04 [-0.18, 0.10]
Suspected	3.50 [2.61, 4.57]	0.21 [-0.11, 0.52]	-90.12 [-154.17, -40.77]	0.08 [-0.22, 0.40]	Suspected	4.81 [3.74, 5.99]	0.18 [-0.18, 0.48]	-23.12 [-45.0, -11.92]	-0.04 [-0.39, 0.30]
Definite	3.10 [2.43, 3.86]	0.19 [-0.04, 0.41]	-77.11 [-157.29, -35.05]	0.03 [-0.21, 0.25]	Definite	4.55 [3.49, 5.68]	0.11 [-0.35, 0.54]	-31.07 [-56.64, -15.38]	-0.09 [-0.50, 0.40]
Clinical Disorder	4.29 [3.15, 5.52]	0.15 [-0.18, 0.46]	-90.31 [-176.51, -39.13]	0.01 [-0.33, 0.36]	Clinical Disorder	3.71 [2.35, 5.01]	-0.12 [-0.55, 0.32]	-18.40 [-55.84, -4.62]	-0.38 [-0.72, 0.04]

Table S5. Brain-age prediction accuracy within PE groups for the two ALSPAC imaging waves (raw predictions). Columns give the MAE, the Pearson correlation between chronological age and predicted age (r Age-pAge), the coefficient of determination (R^2), the correlation between the brainPAD and chronological age (r BrainPAD-Age) followed by the 95 % bootstrap confidence interval, (5000 resamples). Because both waves span very narrow age intervals (19–22 y in MRI-I and 27–32 y in MRI-II), the variance of chronological age is small; as a consequence the model can explain less variance than a naïve "group mean" predictor, yielding negative R^2 values or low correlation between real age and predictions even when MAE indicate reasonable performance.

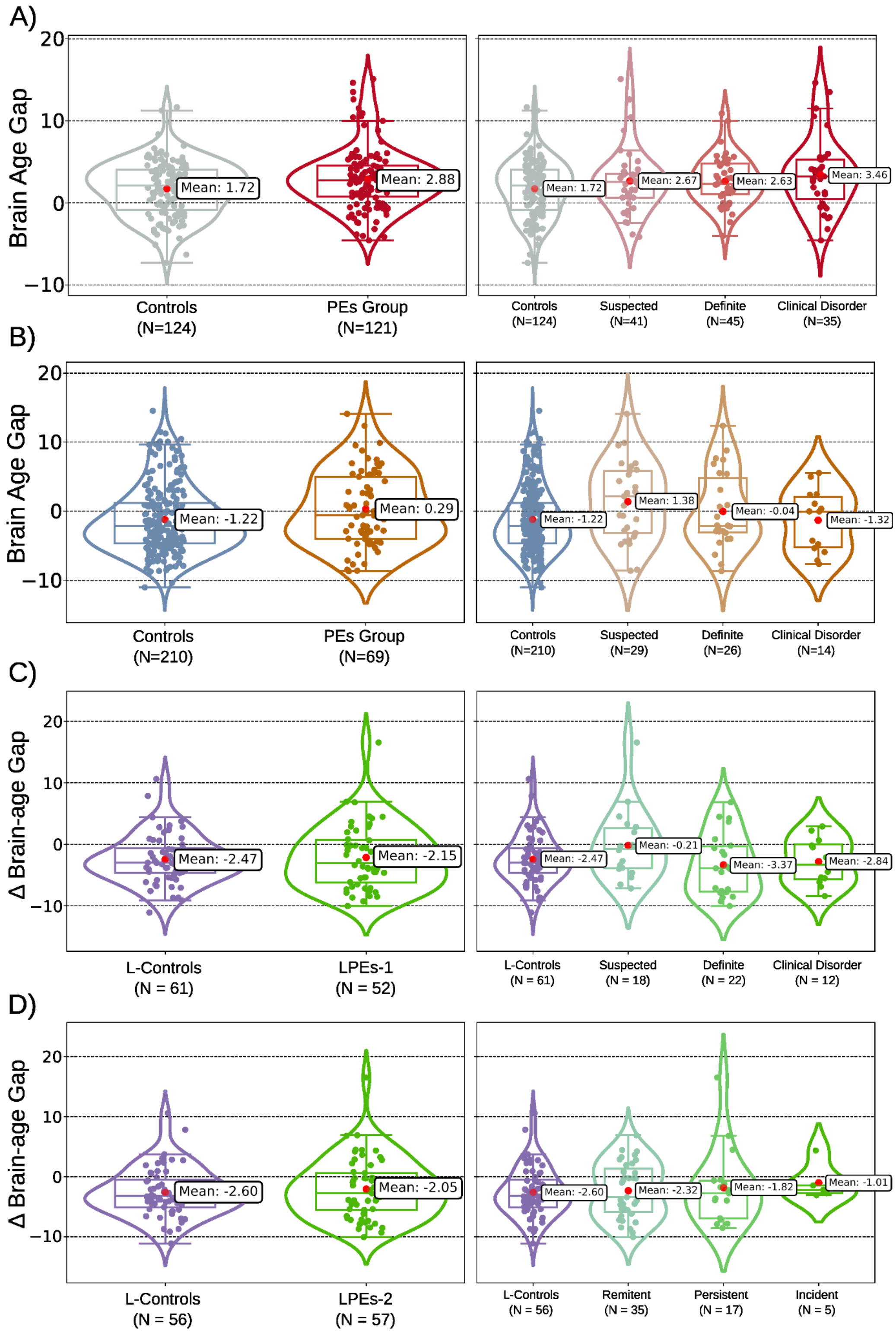

Figure S2. Rain-cloud plots of the uncorrected brainPAD for each hypothesis. A) Baseline (ALSPAC 20 MRI-I). Left panel BrainPAD in controls vs participants with any psychotic experience (PE+). Right panel: BrainPAD across the three PE-severity levels defined by PLIKS-18 (suspected, definite, clinical disorder). B) Follow-up (ALSPAC 30 MRI-II). Left: PE+ vs controls; right: PE-severity split as in A. C) Longitudinal contrasts, LPEs-1 definition (baseline PE status). Left: PE+ vs controls; right: severity split. D) Longitudinal contrasts, LPEs-2 definition (symptom trajectory). Left: controls vs any PE; right: Remitted, Persistent and Incident subgroups. Box widths are proportional to sample size; gray density curves depict the full distribution.

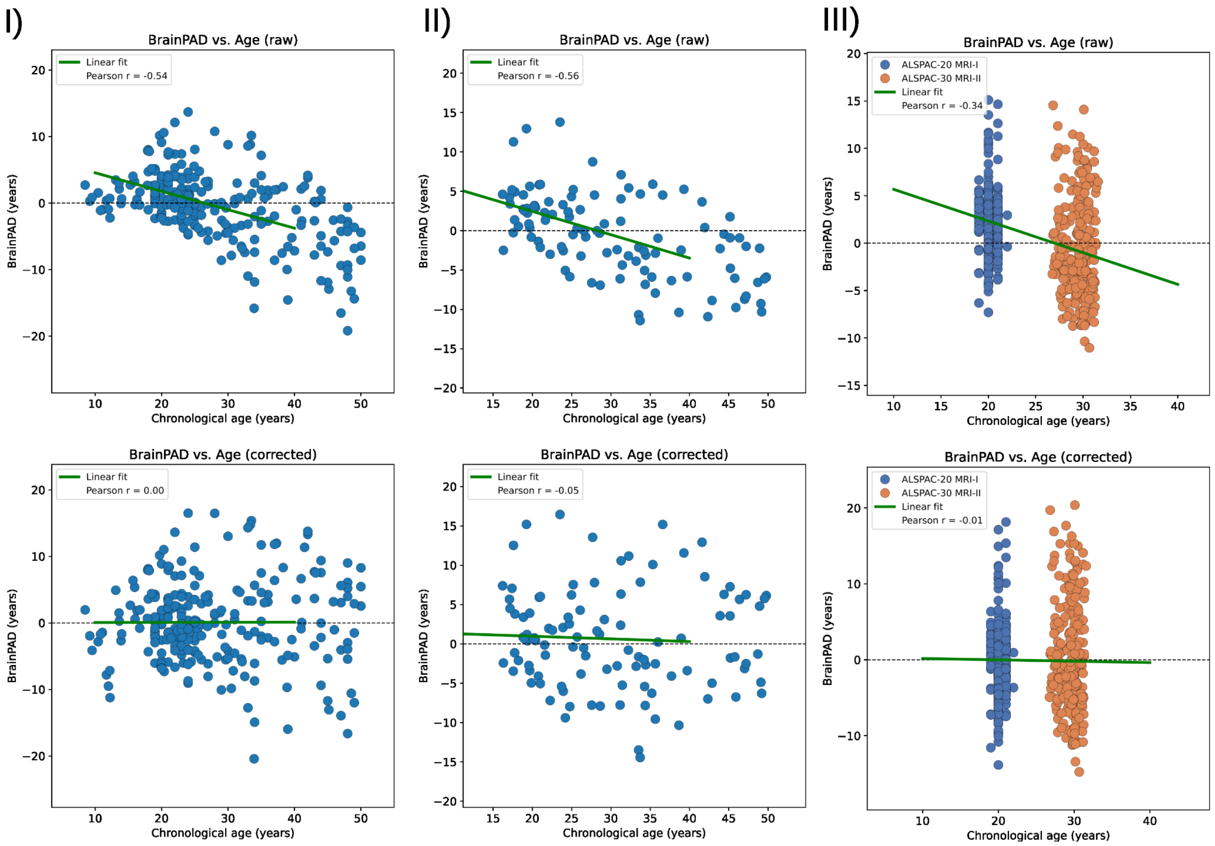

Figure S3. Removal of chronological-age bias from the brainPAD. Each column displays the brainPAD versis chronological age before bias correction (top row) and after correction (bottom row). Green lines are linear fits; Legend shows Pearson's r. I) Internal hold-out-test. The raw brainPAD exhibits a strong negative age trend (r = -0.54). After applying the linear bias correction learned on the validation test, r = 0.00. II) External test set (AgeRisk). A similar raw bias is observed (r = -0.56) and is again removed by the fixed correction (r = -0.05). III) ALSPAC application cohort (19-32 years; blue = ALSPAC-20, MRI-I, orange = ALSPAC-30 MRI-II). Even within this narrow age window the uncorrected brainPAD still correlates with age (r = -0.34). The derived correction reduces the correlation to virtually zero (r = -0.01).

Model results after age-bias correction

PLIKS-18	MAE	r Age-pAge	R^2	r BrainPAD-Age	PLIKS-18	MAE	r Age-pAge	R^2	r BrainPAD-Age
ALSPAC 20 MRI-I					ALSPAC 30 MRI-II				
Controls	3.65 [3.14, 4.17]	0.05 [-0.12, 0.22]	-52.88 [-79.14, -35.29]	-0.08 [-0.26, 0.09]	Controls	5.40 [4.86, 5.93]	0.20 [0.06, 0.33]	-32.68 [-41.81, -25.45]	0.03 [-0.11, 0.16]
Suspected	3.93 [2.79, 5.22]	0.20 [-0.1, 0.51]	-124.96 [-224.06, -56.26]	0.11 [-0.18, 0.43]	Suspected	6.81 [5.24, 8.55]	0.18 [-0.18, 0.50]	-46.24 [-93.47, -26.26]	0.02 [-0.34, 0.38]
Definite	3.22 [2.47, 4.06]	0.19 -0.04, 0.40]	-81.78 [-193.35, -38.55]	0.07 [-0.16, 0.29]	Definite	6.06 [4.41, 7.95]	0.09 [-0.34, 0.54]	-55.33 [-112.19, -29.22]	-0.04 [-0.46, 0.43]
Clinical Disorder	4.89 [3.59, 6.37]	0.16 [-0.18, 0.47]	-114.62 [-244.45, -51.98]	0.06 [-0.29, 0.39]	Clinical Disorder	5.11 [3.63, 6.56]	-0.11 [-0.57, 0.32]	-22.98 [-88.53, -9.28]	-0.31 [-0.70, 0.10]

Table S6. summarises brain-age prediction performance after age-bias correction, stratified by PLIKS-18 psychotic-experience severity across the two ALSPAC imaging waves. Metrics are reported separately for the age-20 scan (MRI-I) and the age-30 scan (MRI-II): *MAE*, the *r* and R^2 between chronological and predicted age, and the residual correlation between BrainPAD and chronological age (BrainPAD–Age r) are reported with 95 % bootstrapped confidence intervals (repetitions = 5000).

	Group	Shapiro-Wilk	Levene's Test	ANOVA/ T-test or variants	Xi^2
**First Timepoint**	Control	stat = 0.78, $P < 1x10^{-3}$			
	PEs	stat = 0.76, $P < 1x10^{-3}$	stat = 0.13, P = .715	MWU, stat = 0.76, P = .385	stat = 1.42, P = .233
	Suspected	stat = 0.64, $P < 1x10^{-3}$			
	Definite	stat = 0.64, $P < 1x10^{-3}$			
	Clinical Disorder	stat = 0.77, $P < 1x10^{-3}$	stat = 0.18, P = .160	KW, stat = 27.62, $P < 1x10^{-3}$	stat = 1.69, P = .430
**Second Timepoint**	Control	stat = 0.96, $P < 1x10^{-3}$			
	PEs	stat = 0.94, $P < 1x10^{-3}$	stat = 0.32, P =.571	MWU, stat=0.01, P = .928	stat = 8.29, P = .004
	Suspected	stat = 0.93, P = .440			
	Definite	stat = 0.94, P = .130			
	Clinical Disorder	stat = 0.95, P = .570	stat = 0.40, P = .753	KW, stat=0.18, P = .192	stat = 3.07, P = .216
**Longitudinal cohort - LPEs-1**	Longitudinal Controls	stat = 0.77, $P < 1x10^{-3}$			
	PEs	stat = 0.79, $P < 1x10^{-3}$	stat=0.63, P = .634	MWU, stat=0.47, P = .494	stat = 0.00, P = .999
	Suspected	stat = 0.64, $P < 1x10^{-3}$			
	Definite	stat = 0.52, $P < 1x10^{-3}$			
	Clinical Disorder	stat = 0.82, P = .180	stat = 0.24, P = .238	KW, stat = 15.04, $P < 1x10^{-3}$	stat=1.34, P = .513
**Longitudinal cohort - LPEs-2**	Longitudinal Controls	stat = 0.78, $P < 1x10^{-3}$			
	PEs	stat = 0.78, $P < 1x10^{-3}$	stat = 0.01, P = .912	MWU, stat = 0.43, P = .514	stat = 0.00, P = .999
	Non-persistent	stat = 0.80, $P < 1x10^{-3}$			
	Persistent	stat = 0.68, $P < 1x10^{-3}$			
	Incident	stat = 0.55, $P < 1x10^{-3}$	stat = 1.46, P = .231	KW, stat = 0.98, P = .322	stat = 0.06, P = .811

Table S7. Shows the test to determine normality and equality of variances among the groups compared for the pairwise comparisons and the trend analysis.

Brain Age model - LMM - diagnostics

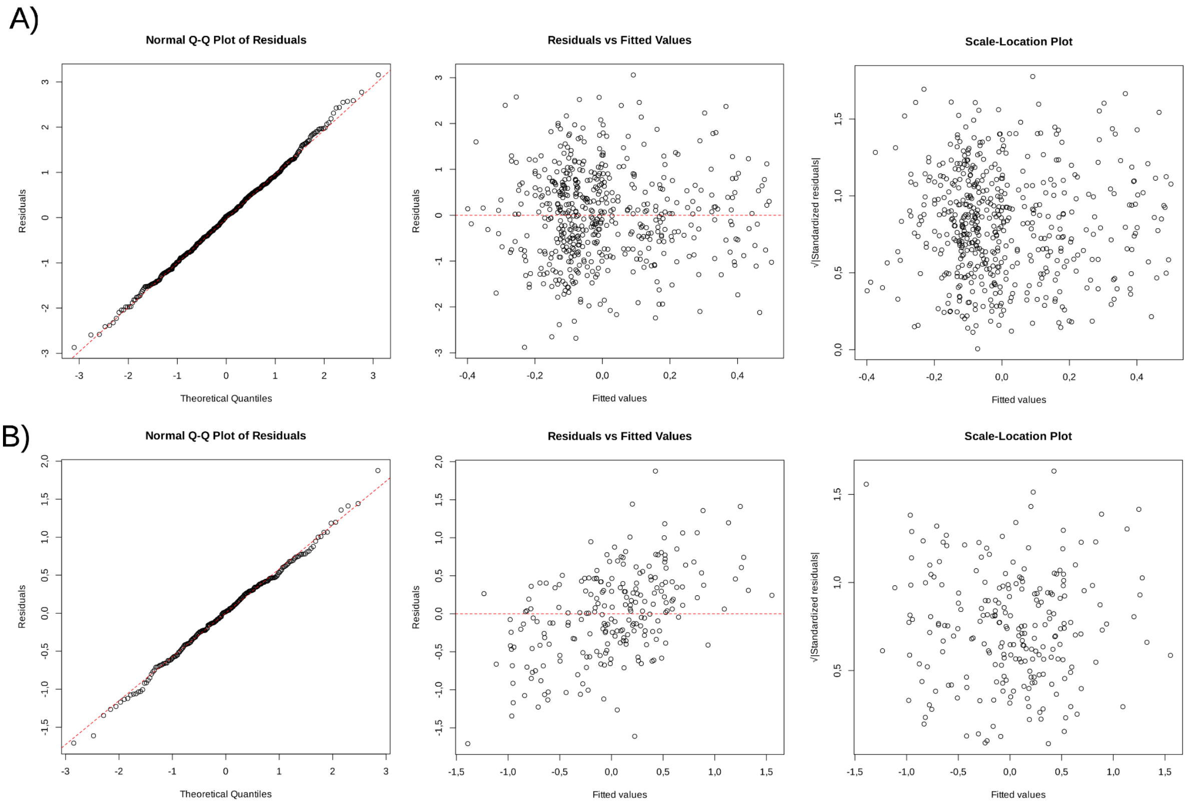

Figure S4. Diagnostic plots for the two linear mixed-effects models. A) LMM defined with LPEs-1. The Q-Q plot follows the 45° line closely, indicating approximate normality. The residuals-versus-fitted and scale–location panels display a roughly constant spread, confirming that the group-specific plus exponential variance structure used in the final model has removed the heteroscedasticity detected in the initial fit. B) LMM defined with LPEs-2. Residuals again adhere to normality, and neither the residuals-versus-fitted nor the scale–location plot shows a systematic "fan" or trend. Because homoscedasticity is satisfied, the simpler random-intercept specification was retained for this outcome.

Contrast	ALSPAC 20 MRI-I — d [95 % CI]	*q*	ALSPAC 30 MRI-II — d [95 % CI]	*q*
Controls – Suspected	-0.25 [-0.61, 0.11]	.344	-1.06 [-2.00, -0.12]	.160
Controls – Definite	-0.27 [-0.59, 0.05]	.296	-0.46 [-1,31, 0.40]	.476
Controls – Clinical Disorder	-0.43 [-0.85, -0.02]	.247	0.09 [-1.22, 1.40]	.896
Suspected – Definite	-0.02 [-0.44, 0.39]	.924	0.61 [-0.59, 1.81]	.476
Suspected – Clinical Disorder	-0.19 [-0.68, 0.31]	.586	1.15 [-0.41, 2.71]	.440
Definite – Clinical Disorder	-0.17 [-0.63, 0.30]	.586	0.54 [-0.96, 2.04]	.571

Table S8. Cohen's *d* (with 95 % confidence intervals) is shown for every pair of PE categories at the 20-year scan (ALSPAC-20 MRI-I) and the 30-year scan (ALSPAC-30 MRI-II). P-values are corrected for multiple comparisons by Benjamini–Hochberg.

Brain Age model - LMM - Results

Effect	β (SE)	*P*
Intercept	−0.123 (0.095)	.196
Time	0.101 (0.112)	.370
Sex	-0.088 (0.090)	.332
Euler n	0.002 (0.002)	.267
Suspected vs Controls	0.252 (0.184)	.172
Definite vs Controls	0.272 (0.165)	.099
**Clinical Disorder vs Controls**	**0.439 (0.214)**	**.041**
Time1:Suspected	0.233 (0.285)	.415
Time1:Definite	-0.065 (0.255)	.800
Time1:Clinical Disorder	-0.478 (0.369)	.198

Hypothesis	Contrast	Δ	*P*	*q*	Cohen's *d* [95 % CI]
H1 – Cross-sectional gap	20 y: Controls vs PEs	0.32 ± 0.13	**.014**	**.029**	**0.70 [0.14, 1.27]**
	30 y: Controls vs PEs	0.22 ± 0.15	.153	.153	0.22 [-0-08, 0.51]
H2 – Severity trend	20 y: linear contrast	1.34 ± 0.68	.049	.098	1.32 [0.00, 2.64]
	30 y: linear contrast	-0.40 ± 0.95	.677	.677	-0.39 [-2.25, 1.47]
H3 – Longitudinal divergence	LPEs-1 (Δ Controls – Δ PEs)	-0.10 ± 0.20	.604	.604	-0.23 [-0.79, 0.33]
	LPEs-2 (Δ Controls – Δ PEs)	0.07 ± 0.22	.751	.751	0.10 [-0.36, 0.51]

Table S9. Summary of fixed effects and planned contrasts from the primary linear mixed-effects model. The left block lists the regression coefficients (β) with their standard errors (SE) and p-values for each fixed effect. The right block shows the specified hypotheses (H_1–H_3), the corresponding contrasts, the estimated differences in brainPAD (corrected and Yeo-Johnson power transformed, Δ ± SE), their raw *p*-values, Benjamini–Hochberg adjusted *q*-values, and the standardised effect sizes (Cohen's *d*) with 95 % confidence intervals (CI).

**Effect**	**β (SE)**	***P***
Intercept	-0.129 (0.140)	.359
Time	0.194 (0.133)	.147
Sex	-0.058 (0.164)	.724
Euler n	-0.002 (0.002)	.389
remitted vs L-Controls	0.119 (0.206)	.564
Persistent vs L-Controls	0.100 (0.266)	.707
Incident vs L-Controls	0.318 (0.447)	.478
Time:remitted	-0.045 (0.215)	.835
Time:Persistent	0.054 (0.276)	.845
Time:Incident	0.203 (0.465)	.663

Table S10. Fixed-effect coefficients for the second linear mixed-effects model (LPEs-2).

Contrast	d [CI 95 %]	*q*
ΔL-Controls− ΔSuspected	-0.23 [-0.79, 0.33]	.416
ΔL-Controls− ΔDefinite	0.06 [-0.44, 0.57]	.800
ΔL-Controls− ΔClinical Disorder	0.47 [-0.25, 1.20]	.198
ΔSuspected − ΔDefinite	0.29 [-0.39, 0.98]	.400
ΔSuspected − ΔClinical Disorder	0.70 [-0.16, 1.56]	.108
ΔDefinite − ΔClinical Disorder	0.41 [-0.42, 1.23]	.865
L-Controls - Remitted	0.06 [-0.76, 0.88]	.845
L-Controls - Persistent	-0.08 [-1.13, 0.98]	.845
L-Controls - Incident	-0.29 [-2.06, 1.49]	.845
Remitted - Persistent	-0.14 [-1.27, 0.99]	.845
Remitted - Incident	-0.35 [-2.17, 1.46]	.845
Persistent - Incident	-0.21 [-2.15, 1.72]	.845

Table S11. Pairwise contrasts of longitudinal change (Δ) with the LPEs-1 definition (top table) and the LPEs-2 definition (bottom table). Entries show Cohen's *d* with its 95 % confidence interval and the *q*-value after false-discovery-rate correction (Benjamini–Hochberg). A positive *d* indicates that the first-listed group experienced a larger increase (or a smaller decrease) than the second (L-Controls, Longitudinal controls).

Euler number sensitivity analysis

To assess the impact of segmentation quality on our findings, we reran every model after excluding the 5 % of scans with the lowest Euler numbers, retaining 499 of 524 observations. Because the two imaging waves used different MRI protocols (ALSPAC-20 MRI-I: SPGR; ALSPAC-30 MRI-II: MPRAGE), Euler numbers were first standardised to ensure they were comparable across protocols. At age 20 the Control-versus-PE difference changed from 0.32 ± 0.13 (*d* [95 % CI] = 0.70 [0.14, 1.27]; $q = .029$) to 0.30 ± 0.13 (*d* [95 % CI] = 0.66 [0.09, 1.23]; $q = .048$) the point estimate moved by -0.02. The linear trend across the four PLIKS levels at age 20 was still positive ($\beta = 1.27$) but lost nominal significance before FDR correction ($q = .070$ from $P = .049$); its magnitude differed by - 0.07. from the original estimate. The ALSPAC 30 MRI-II trend and all other contrasts stayed non-significant. Complete results for the sensitivity analysis can be found in Table S12 and Table S13.

Effect	β (SE)	*p*
Intercept	−0.126 (0.097)	.196
Time	0.132 (0.115)	.255
Sex	-0.121 (0.092)	.191
Euler n	0.003 (0.002)	.098
Suspected vs Controls	0.258 (0.183)	.160
Definite vs Controls	0.209 (0.167)	.213
Clinical Disorder vs Controls	0.439 (0.222)	.049
Time1:Suspected	0.225 (0.303)	.460
Time1:Definite	0.024 (0.264)	.923
Time1:Clinical Disorder	−0.510 (0.367)	.168

Hypothesis	Contrast	Δ	*P*	*q*	Cohen's *d* [95 % CI]
H1 – Cross-sectional gap	20 y: Controls vs PEs	0.30 ± 0.13	**.024**	**.048**	**0.66 [0.09, 1.23]**
	30 y: Controls vs PEs	0.22 ± 0.15	.167	.167	0.23 [-0.10, 0.55]
H2 – Severity trend	20 y: linear contrast	1.27 ± 0.70	.070	.140	1.34 [-0.11, 2.79]
	30 y: linear contrast	-0.46 ± 0.93	.621	.621	-0.49 [-2.44, 1.46]
H3 – Longitudinal divergence	LPEs-1 (Δ Controls – Δ PEs)	-0.09 ± 0.20	.670	.574	-0.19 [-0.57, 0.19]
	LPEs-2 (Δ Controls – Δ PEs)	0.13 ± 0.22	.574	.574	0.18 [-0.19, 0.55]

Table S12. Fixed-effect results for the first linear mixed-effects model (LPEs-1). The 5 % of scans with the poorest Euler numbers (25 of 524 scans) were discarded, leaving 499 scans. Model diagnostics: residual normality (Shapiro–Wilk W = 1.00, *P* = .950) and homoscedasticity (Breusch–Pagan BP = 0.32, *P* = 0.574) were both satisfactory.

Effect	β (SE)	*P*
Intercept	-0.129 (0.140)	.359
Time	0.194 (0.133)	.147
Sex	-0.058 (0.164)	.724
Euler n	-0.001 (0.002)	.389
remitted vs L-Controls	0.119 (0.206)	.564
Persistent vs L-Controls	0.100 (0.266)	.707
Incident vs L-Controls	0.318 (0.447)	.478
Time:remitted	-0.045 (0.215)	.835
Time:Persistent	0.054 (0.276)	.845
Time:Incident	0.203 (0.465)	.663

Table S13. Fixed-effect results for the second linear mixed-effects model (LPEs-2). This model compares longitudinal brainPAD change across four clinical trajectories, longitudinal controls, remitted, persistent and incident psychotic experiences, using the same covariates, as LMM 1 and applying the same 5 % Euler-number rule. Model diagnostics: residual normality (Shapiro–Wilk W = 1.00, P = .878) and homoscedasticity (Breusch–Pagan, BP = 0.06, P = .813) were both satisfied.

Depression sensitivity analysis

Mixed-effects models were re-estimated to test whether the association between PEs and brainPAD was independent of depressive morbidity. Complete data on both ICD-10 depression items were available for $N = 379$ individuals, with 484 scans. A total of 105 individuals had scans at both timepoints. Distribution of depressive symptoms across timepoints and PEs levels can be found in Table S14.

After restricting the analysis to participants with complete data (N = 484), adding the depression main effect and its interaction with PEs level, the Akaike Information Criterion (AIC) raised from 1407.66 to 1413.58, modestly worsening fit. The depression score had a small influence on brainPAD (*d* [95 % CI] = 0.23 [0.03, 0.43], $P = .027$, uncorrected), yet the PE effects persisted. The comparison was not corrected since it was an exploratory analysis. At baseline (ALSPAC 20 MRI-I), the combined PE group remained significantly older in brain-age than controls (*d* [95 % CI] = 0.69 [0.16, 1.23], $q = .022$); At follow-up (ALSPAC 30 MRI-II), the control-versus-PE contrast was nonsignificant and small (*d* [95 % CI] = 0.14 [-0.18, 0.46], $q = .391$). Furthermore, in H2, trend analysis for the first acquisition was not significant but showed a large effect (*d* [95 % CI] = 1.32 [-0.18, 2.82], $q = .168$) while it was non-significant and with a small effect for the second time-point (*d* [95 % CI] = -0.40 [-2.50, 1.69], $q = .704$). Finally, in the longitudinal subsample, adding depression did not alter any interaction, regardless of whether PE trajectory was coded as LPEs-1 or LPEs-2, with all interaction *q*-values > 0.60. Nevertheless, for the second LLM AIC was reduced from 618.90 without the depression covariate and interaction to 586.90 when including the depression score. The fitted model with the LPEs-1 definition retained normal and homoscedastic residuals (Shapiro–Wilk, $W = 1.00$, $P = .978$; Breusch–Pagan, $BP = 0.73$, $P = .392$). This was the same in the second model using the LPEs-2 definition (Shapiro–Wilk, $W = 1.00$, $P = .921$; Breusch–Pagan, $BP = 0.002$, $P = .967$). Full results of the LMMs fitted can be found in Table S15 and Table S16.

Time	pliks18TH	No Symptoms or diagnosis	Symptoms or Diagnosis	Symptoms and Diagnosis	Total
ALSPAC 20 MRI-I	Controls	101 (84.87 %)	7 (5.88 %)	11 (9.24 %)	119
	Suspected	26 (72.22 %)	4 (11.11 %)	6 (16.67 %)	36
	Definite	29 (69.05 %)	2 (4.76 %)	11 (26.19%)	42
	Clinical Disorder	16 (50.00 %)	3 (18.75 %)	13 (40.63 %)	32
ALSPAC 30 MRI-II	Controls	177 (90.08 %)	7 (3.59 %)	11 (5.64 %)	195
	Suspected	20 (83.33 %)	0 (0.00 %)	4 (16.67 %)	24
	Definite	18 (78.25 %)	1 (4.35 %)	4 (17.40 %)	23
	Clinical Disorder	6 (46.15 %)	1 (7.70 %)	6 (46.15 %)	13
Total		393 (81.20 %)	25 (51.65 %)	66 (13.64 %)	484

Table S14. Distribution of 17-year depression status across PEs categories (LPEs-1) at the two MRI waves. For each level, Controls, Suspected, Definite and Clinical Disorder, the table shows the number of participants and the within-row percentage who had (i) no depressive symptoms or diagnosis, (ii) subthreshold symptoms or an ICD-10 diagnosis, and (iii) both symptoms and an ICD-10 diagnosis at age 17. Counts are reported separately for the first scan (ALSPAC-20, baseline) and the second scan a decade later (ALSPAC-30, follow-up), along with column totals for the pooled sample (N = 484). Percentages are calculated within each pliks18TH row.

Effect	β (SE)	*P*
Intercept	–0.210 (0.099)	.035
Time	0.167 (0.113)	.142
Sex	–0.066 (0.093)	.474
Euler n	0.001 (0.002)	.711
**Depression**	**0.227 (0.102)**	**.027**
Suspected vs Controls	0.260 (0.213)	.222
**Definite vs Controls**	**0.461 (0.182)**	**.012**
Clinical Disorder vs Controls	0.492 (0.281)	.081
Suspected: Depression	0.140 (0.220)	.526
**Definite: Depression**	**–0.430 (0.168)**	**.011**
Clinical Disorder: Depression	–0.138 (0.211)	.513
Time1:Suspected	0.046 (0.305)	.880
Time1:Definite	–0.172 (0.259)	.508
Time1:Clinical Disorder	–0.518 (0.398)	.197

Hypothesis	Contrast	Δ	*P*	*q*	Cohen's *d* [95 % CI]
H1 – Cross-sectional gap	20 y: Controls vs PEs	0.36 ± 0.14	**.011**	**.022**	**0.69 [0.16, 1.23]**
	30 y: Controls vs PEs	0.14 ± 0.17	.391	.391	0.14 [-0.18, 0.46]
H2 – Severity trend	20 y: linear contrast	1.36 ± 0.78	.084	.168	1.32 [-0.18, 2.82]
	30 y: linear contrast	-0.42 ± 1.09	.704	.704	-0.40 [-2.50, 1.69]
H3 – Longitudinal divergence	LPEs-1 (Δ Controls – Δ PEs)	-0.22 ± 0.21	.309	.618	-0.42 [-1.22, 0.39]
	LPEs-2 (Δ Controls – Δ PEs)	0.08 ± 0.23	.733	.733	0.11 [-0.37, 0.53]

Table S15. Fixed-effect results for the first linear mixed-effects model (LPEs-1). The depression covariate and its interactions with PLIKS were included in the model. Model diagnostics: residual normality (Shapiro–Wilk W = 1.00, *P* = .978) and homoscedasticity (Breusch–Pagan BP = 0.73, *P* = .392) were both satisfactory.

Effect	β (SE)	*p*
Intercept	–0.184 (0.153)	.230
Time	0.211 (0.138)	.129
Sex	–0.026 (0.177)	.883
Euler n	–0.002 (0.002)	.369
Depression	0.278 (0.209)	.185
remitted vs L-Controls	0.116 (0.237)	.623
Persistent vs L-Controls	0.161 (0.321)	.617
Incident vs L-Controls	–0.025 (0.604)	.967
Time:remitted	–0.084 (0.228)	.712
Time:Persistent	0.133 (0.287)	.644
Time:Incident	0.185 (0.470)	.694
remitted: depression	–0.141 (0.276)	.610
Persistent: depression	–0.306 (0.294)	.301
Incident: depression	0.206 (0.534)	.700

Table S16. Fixed-effect coefficients for the second linear mixed-effects model (LPEs-2). The depression score and its interactions with PLIKS levels were included in the model. Model diagnostics: residual normality (Shapiro–Wilk W = 1.00, *P* = .921) and homoscedasticity (Breusch–Pagan BP = 0.002, P = .967) were both satisfied.

Longitudinal reliability of brain-age estimates

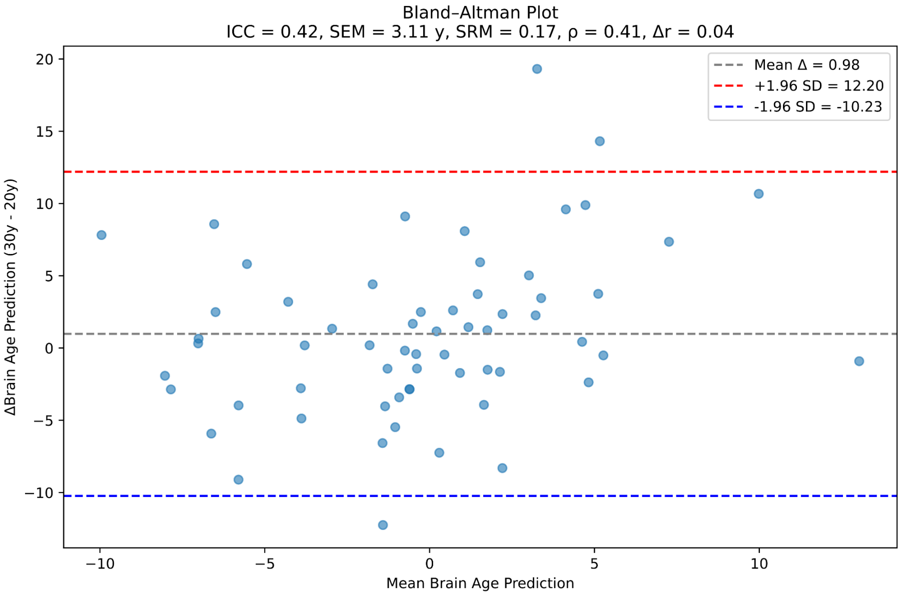

Figure S5. Bland–Altman plot shows the within-subject difference in brainPAD between the 30-year scan (age range 27–32 years) and the 20-year scan (age range 19–22 years) against the mean of the two observations. The dashed gray line marks the mean difference (Δ = 0.01 years); outer dashed lines indicate the ±1.96 SD limits of agreement (LoA ≈ ±6.0 years). ICC (3,1) [95 % CI] = 0.42 [0.19, 0.60], SEM = 3.11 years. Spearman ρ = 0.41; concordance correlation coefficient = 0.41 (0.16–0.63). Δ Brain-age correlates negligibly with Δ Age (r = 0.04); a mixed-effects slope of 1.01 ± 0.08 confirms that predicted age keeps pace with chronological age across the ≈10-year interval. These results indicate minimal systematic bias and fair long-term reliability: individual brain-age estimates remain on average stable over a decade, but the error magnitude (~3 years) approaches the natural age spread within each scan wave, limiting sensitivity to small longitudinal changes.

Model-free validation analysis

To verify that our findings are not artifacts of supervised ML modeling or the harmonization pipeline, we performed two unsupervised checks. We repeated all analyses on unharmonized features and on ComBat-GAM–harmonized features to test sensitivity to harmonization choices.

A) Control-anchored PCA: age-linked components: Because each wave has a short age range, we pooled strict longitudinal controls (PLIKS=0 at both waves) to widen the normative age range. In controls only, we fit a PCA on the full feature set (50 PCs). For each PC we computed Spearman correlation with age in controls and oriented the sign so higher scores are 'older-like'. We focused on the most age-sensitive PC and, as sensitivity, inspected the next four. To quantify scanner effects (controls, per PC), we fit OLS models on the oriented raw $PC \sim age+age^2$ (report R^2(Age)) and $PC \sim age+age^2+Scanner$ (report R^2(+Scanner) and ΔR^2), and we reported the partial Pearson r(Age|Scanner) to index the within-scanner age signal. To isolate deviations from normative aging, each PC was regressed on age and age^2 in controls. That model was applied to all participants, and the residuals were related to PLIKS at MRI-I and MRI-II using Spearman correlation. We repeated the entire pipeline on unharmonized and ComBat-GAM–harmonized features.

B) Longitudinal magnitude of change: For each paired subject, we computed annualized percent change (APC) for every feature between waves. We summarised within-subject longitudinal change as the mean absolute APC per year across feature and tested its association with PLIKS-18 using Spearman correlation and OLS.

For the unsupervised approach (A), all results per-component are in Table S17 (below). With the most age-sensitive PC:

- Unharmonized (PC1). MRI-I: $\rho = 0.124$, $P = 0.054$. MRI-II: $\rho = 0.122$, $P = 0.041$
- Harmonized (PC0). MRI-I: $\rho = 0.141$, $P = 0.027$. MRI-II: $\rho = 0.147$, $P = 0.014$

After ComBat-GAM harmonization, the control-trained age axis (PC0) shows a small, consistent positive link between PLIKS and older-like scores at both waves ($\rho \sim 0.14$), with a strong within-scanner age signal and no added scanner variance ($\Delta R^2 = 0$). In unharmonized data, K=1 shows the same positive direction but weaker ($\rho \sim 0.12$) and clearly confounded by scanner ($\Delta R^2 = 0.07$; partial r(Age|Scanner) = −0.201), other unharmonized PCs are mixed. These results suggest that higher PLIKS relates to slightly older-than-expected anatomy, with modest

effect sizes. Cumulative explained variance of the selected K=5 PCs is ~39% (unharmonized) and ~29% (harmonized). PCs were chosen by age-correlation, not by variance.

PC	EVR	ρ(age) in controls	MRI-I ρ (p)	MRI-II ρ (p)	R²(Age)	R²(Scanner)	ΔR²	partial r(Age|Scanner) (p)
**Harmonization**								
PC1	0.101	0.630	0.124 (0.053)	0.122 (0.041)	0.551	0.621	0.070	-0.201 (0.034)
PC0	0.179	0.582	-0.120 (0.061)	-0.142 (0.018)	0.346	0.356	0.010	0.184 (0.053)
PC2	0.073	0.151	-0.027 (0.676)	0.117 (0.052)	0.022	0.071	0.049	0.265 (0.005)
PC4	0.029	0.135	-0.117 (0.066)	-0.083 (0.167)	0.007	0.099	0.093	0.273 (0.004)
PC43	0.005	0.129	-0.001 (0.991)	-0.005 (0.931)	0.002	0.048	0.047	0.203 (0.033)
**Harmonized (ComBat-GAM)**								
PC0	0.201	0.342	0.141 (0.027)	0.147 (0.014)	0.220	0.220	0	0.469 (1.85e-07)
PC21	0.012	0.252	-0.132 (0.040)	-0.112 (0.062)	0.044	0.044	0	0.210 (0.026)
PC2	0.045	0.231	-0.072 (0.260)	-0.114 (0.057)	0.058	0.058	0	0.217 (0.021)
PC30	0.008	0.198	-0.008 (0.901)	0.087 (0.148)	0.034	0.034	0	0.171 (0.071)
PC8	0.021	0.189	-0.005 (0.936)	-0.080 (0.180)	0.027	0.027	0	0.164 (0.084)

Table S17. Per-component associations between a control-trained PCA "age axis" and PLIKS-18, before and after harmonization. PCA was fit only on strict longitudinal controls (PLIKS = 0 at both waves), and components were oriented so higher scores indicate "older-like" anatomy. For each PC we report: EVR (explained variance ratio in controls), ρ(age) in controls (Spearman correlation on oriented raw PC scores), MRI-I/II ρ (p) (Spearman correlation between PLIKS and the age-adjusted PC residuals at each wave; residuals from a control-trained quadratic model PC~age+age^2; R2(Age) from the control-only quadratic model, R2(+Scanner) after adding a scanner indicator (controls), ΔR^2, incremental variance explained by Scanner, and partial r(Age|Scanner)(p) (Pearson partial correlation between age and the PC within scanner, computed in controls). In the harmonized data (ComBat-GAM), adding Scanner does not increase fit ($\Delta R^2 \approx 0$ across PCs), while the within-scanner age signal remains strong for PC0 (partial r = 0.469). In the unharmonized data, several PCs, especially PC1, show meaningful Scanner-added variance (e.g., $\Delta R^2 = 0.07$) and weak/negative within-scanner age correlation (partial r = 0.201), indicating that the large pooled age association is largely between-scanner.

Harmonization makes scanners comparable, so the main age pattern moves from unharmonized PC1 (ρ = 0.630) to the harmonized PC0 (ρ = 0.342) and the PC order changes. After harmonization, within each scanner the PC still goes up with age (partial r = 0.469). In the raw (unharmonized) data, PC1 shows the opposite within-scanner pattern (partial r=−0.201), which suggests part of the pooled age effect there comes from differences between scanners rather than a clean age signal. The unharmonized PC0 explains substantial variance (EVR = 0.179) and relates to age in controls (ρ = 0.582). After removing the control-trained age curve, its PLIKS associations are small and negative (Wave I −0.120; Wave II −0.142). Scanner contributes for this PC ($\Delta R^2 = 0.010$) and the within-scanner age link is weakly positive (partial r = 0.184, p = 0.053), so we do not read the negative PLIKS as a strong 'younger-like' effect, it may reflect residual non-age variation.

Analyzing the annualized percent changes (2) per ROIs, there was no evidence that higher PLIKS is associated with a different rate of structural change between scans:

- Unharmonized: ρ = −0.097, *P* = 0.306; OLS β = −0.015 ± 0.029.
- Harmonized: ρ = −0.095, *P* = 0.317; OLS β = −0.008 ± 0.020.

Taken together, the control-trained PCA checks support the manuscript's main conclusions. In the paper we reported that youths with higher PLIKS show a modestly higher brainPAD at the first acquisition, that this group

difference is not clear at the second acquisition, and that the gap does not diverge across the acquisitions. We fitted PCA in strict controls, removed the control-trained age curve, and related the residual scores to PLIKS. We observe small, consistent positive associations at both waves, most clearly after harmonization. The unharmonized K=1 analysis shows the same direction but weaker. By contrast, longitudinal annualized percent change shows no association with PLIKS (see Figure S6

).

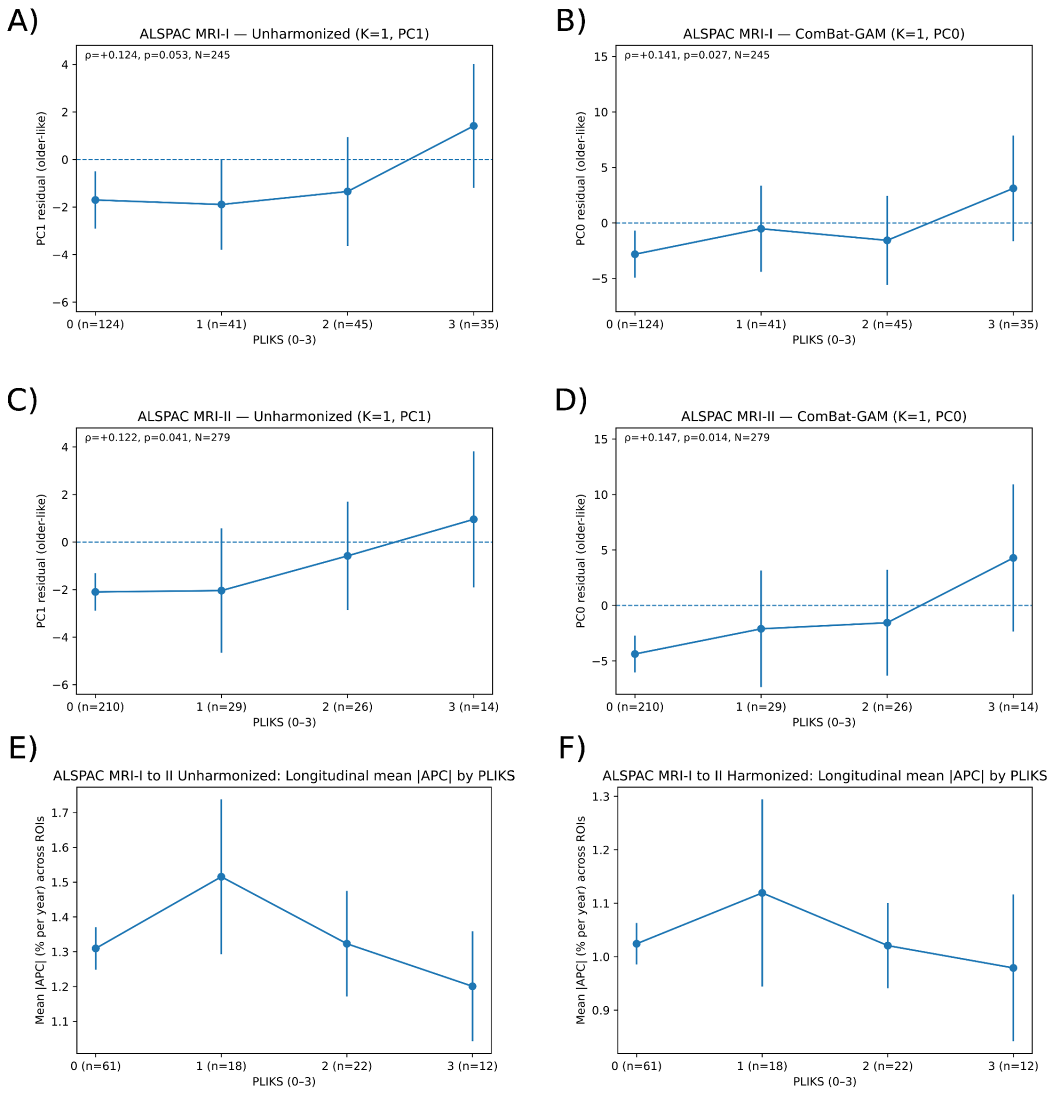

Figure S6. PLIKS dose–response on age-adjusted PCA scores (K=1). Panels show ALSPAC MRI-I (A–B) and MRI-II (C–D), using unharmonized features (left) and ComBat-GAM–harmonized features (right). The age-sensitive component was learned only from strict longitudinal controls: PCA was fit on controls, and the component was oriented so higher values indicate “older-like” anatomy. Within each wave we then removed the normal age pattern (age + age² fitted on controls) and plotted the residual PC score by PLIKS category. In both waves the harmonized K=1 component shows a positive association with PLIKS, with concordant positive trends in the unharmonized data. Panels E–F summarise longitudinal change from MRI-I to MRI-II as mean |APC| per year across ROIs. E uses unharmonized features; F uses ComBat-GAM–harmonized features. In both pipelines there is no evidence that higher PLIKS is associated with a different rate of structural change.

Power analysis

To quantify the sensitivity of our longitudinal mixed–model tests we ran a Monte-Carlo simulation that reproduces our analysis pipeline. Each simulated data set reproduced the observed sample structure: 524 participants in total, of whom 245 were assessed at age-20 and 279 at age-30; 113 individuals contributed data at both waves. The distribution of psychotic-like experiences was fixed to the empirical counts.

Outcome values were generated in standard-deviation (SD) units. At baseline the mean Brain-PAD was set to 0 SD for controls and to 0.30, 0.60 and 0.9 SD for PLIKS levels 1, 2 and 3, respectively. Sex (coded 0 = female, 1 = male) and Euler number were included as covariates with coefficients of +0.10 SD and –0.05 SD per SD of Euler, respectively. Sex and Euler values for each synthetic participant were bootstrapped, with replacement, from their empirical distributions so that the marginal distributions matched the real cohort.

Subject-specific random intercepts and Time slopes were drawn from a bivariate normal distribution with SDs of 1.00 and 0.30 and a correlation of 0.10. Residual variances were allowed to differ by PLIKS level (σ = 1.00, 1.10, 1.15 and 1.20 for levels 0–3). Each simulated data set was analyzed with the exact model used in the study for each LPEs definition and level-specific residual weights. The three families of contrasts were extracted with emmeans:

1. Control vs pooled PLIKS-positive at ALSPAC 20 MRI-I and the same contrast at ALSPAC 30 MRI-II
2. Linear severity trends at each wave per PEs levels
3. The 10-year divergence using LPEs-1 and LPEs-2 definitions

One thousand replicate data sets were generated and analyzed in parallel. Power was estimated as the proportion of replicates with $P < .050$ for each contrast. Table S18, shows the results.

Hypothesis	Description	Power (α = 0.05, n = 1 000)
**H1 base**	Control vs pooled PLIKS 1-3 at age-20	0.900
**H1 follow**	Control vs pooled PLIKS 1-3 at age-30	0.940
**H2 base**	Linear severity trend at age-20	0.909
**H2 follow**	Linear severity trend at age-30	0.873
**H3 LPEs-1**	10-year divergence	0.057
**H3 LPEs-2**	10-year divergence	0.067

Table S18. Under the assumed effect sizes the analysis H1 and H2 enjoyed above 80 % power to detect the baseline severity trend and the raw control-versus-patient gap at both waves. Power was limited for the longitudinal interaction (0.06).

REFERENCES

1. Boyd A, Golding J, Macleod J, Lawlor DA, Fraser A, Henderson J, et al. (2013): Cohort profile: the 'Children of the 90s'—the index offspring of the Avon Longitudinal Study of Parents and Children. Int J Epidemiol 42:111–127.
2. Fraser A, Macdonald-Wallis C, Tilling K, Boyd A, Golding J, Davey Smith G, et al. (2013): Cohort profile: the Avon Longitudinal Study of Parents and Children: ALSPAC mothers cohort. Int J Epidemiol 42:97–110.
3. Northstone K, Lewcock M, Groom A, Boyd A, Macleod J, Timpson N, et al. (2019): The Avon Longitudinal Study of Parents and Children (ALSPAC): an update on the enrolled sample of index children in 2019. Wellcome Open Res 4:51.
4. Wing JK, Babor T, Brugha T, Burke J, Cooper JE, Giel R, et al. (1990): SCAN: schedules for clinical assessment in neuropsychiatry. Arch Gen Psychiatry 47:589–593.
5. Horwood J, Salvi G, Thomas K, Duffy L, Gunnell D, Hollis C, et al. (2008): IQ and non-clinical psychotic symptoms in 12-year-olds: results from the ALSPAC birth cohort. Br J Psychiatry 193:185–191.
6. Zammit S, Kounali D, Cannon M, David AS, Gunnell D, Heron J, et al. (2013): Psychotic experiences and psychotic disorders at age 18 in relation to psychotic experiences at age 12 in a longitudinal population-based cohort study. Am J Psychiatry 170:742–750.
7. Rammos A, Sullivan SA, Kounali D, Jones HJ, Hammerton G, Hines LA, et al. (2022): Precursors and correlates of transient and persistent longitudinal profiles of psychotic experiences from late childhood through early adulthood. Br J Psychiatry 220:330–338.
8. [dataset] Park D, Hennessee J, Smith ET, Chan M, Katen C, Wig G, et al. (2024): The Dallas Lifespan Brain Study. OpenNeuro, version 1.0.4. Available from: https://openneuro.org/datasets/ds004856/versions/1.0.4
9. Zuo XN, Anderson JS, Bellec P, Birn RM, Biswal BB, Blautzik J, et al. (2014): An open science resource for establishing reliability and reproducibility in functional connectomics. Sci Data 1:140049.
10. Spreng RN, Setton R, Alter U, Cassidy BN, Darboh B, DuPre E, et al. (2022): Neurocognitive aging data release with behavioral, structural and multi-echo functional MRI measures. Sci Data 9:119.
11. Marcus DS, Wang TH, Parker J, Csernansky JG, Morris JC, Buckner RL (2007): Open Access Series of Imaging Studies (OASIS): cross-sectional MRI data in young, middle aged, nondemented, and demented older adults. J Cogn Neurosci 19:1498–1507.
12. Wei D, Zhuang K, Ai L, Chen Q, Yang W, Liu W, et al. (2018): Structural and functional brain scans from the cross-sectional Southwest University adult lifespan dataset. Sci Data 5:180134.
13. [dataset] Biomedical Image Analysis Group, Imperial College London (2015): Information eXtraction from Images (IXI) dataset. Available from: https://brain-development.org/ixi-dataset/
14. Shafto MA, Tyler LK, Dixon M, Taylor JR, Rowe JB, Cusack R, et al. (2014): The Cambridge Centre for Ageing and Neuroscience (Cam-CAN) study protocol: a cross-sectional, lifespan, multidisciplinary examination of healthy cognitive ageing. BMC Neurol 14:204.
15. Taylor JR, Williams N, Cusack R, Auer T, Shafto MA, Dixon M, et al. (2017): The Cambridge Centre for Ageing and Neuroscience (Cam-CAN) data repository: structural and functional MRI, MEG, and cognitive data from a cross-sectional adult lifespan sample. Neuroimage 144:262–269.
16. Nugent AC, Thomas AG, Mahoney M, Gibbons A, Smith JT, Charles AJ, et al. (2022): The NIMH intramural healthy volunteer dataset: a comprehensive MEG, MRI, and behavioral resource. Sci Data 9:518.
17. Nárai Á, Hermann P, Auer T, Kemenczky P, Szalma J, Homolya I, et al. (2022): Movement-related artefacts (MR-ART) dataset of matched motion-corrupted and clean structural MRI brain scans. Sci Data 9:630.
18. Nooner KB, Colcombe S, Tobe R, Mennes M, Benedict M, Moreno A, et al. (2012): The NKI-Rockland Sample: a model for accelerating the pace of discovery science in psychiatry. Front Neurosci [Internet] 6. Available from: https://www.frontiersin.org/journals/neuroscience/articles/10.3389/fnins.2012.00152/full
19. McNabb CB, Driver ID, Hyde V, Hughes G, Chandler HL, Thomas H, et al. (2025): WAND: a multi-modal dataset integrating advanced MRI, MEG, and TMS for multi-scale brain analysis. Sci Data 12:220.
20. [dataset] Tisdall L, Mugume S, Kellen D, Mata R (2023): Lifespan trajectories of risk preference, impulsivity, and self-control: a dataset containing self-report, informant-report, behavioral, hormone and functional neuroimaging measures from a cross-sectional human sample. OSF. Available from: https://osf.io/y6jfb
21. Kane LT, Fang T, Galetta MS, Goyal DKC, Nicholson KJ, Kepler CK, et al. (2020): Propensity score matching: a statistical method. Clin Spine Surg 33:120.
22. Henschel L, Conjeti S, Estrada S, Diers K, Fischl B, Reuter M (2020): FastSurfer: a fast and accurate deep learning based neuroimaging pipeline. Neuroimage 219:117012.
23. Alexander B, Loh WY, Matthews LG, Murray AL, Adamson C, Beare R, et al. (2019): Desikan-Killiany-Tourville atlas compatible version of M-CRIB neonatal parcellated whole brain atlas: the M-CRIB 2.0. Front Neurosci [Internet] 13. Available from: https://www.frontiersin.org/journals/neuroscience/articles/10.3389/fnins.2019.00034/full
24. Klein A, Tourville J (2012): 101 labeled brain images and a consistent human cortical labeling protocol. Front Neurosci [Internet] 6. Available from: https://www.frontiersin.org/journals/neuroscience/articles/10.3389/fnins.2012.00171/full
25. [dataset] Zughayyar I, Bauer M, Güttler C, Marcelino A, Kühne F, Buss C, et al. (2025): A FastSurfer database for age-specific brain volumes in healthy children: a tool for quantifying localized and global brain volume alterations in pediatric patients. OSF. Available from: https://osf.io/dw7p4_v3
26. Pomponio R, Erus G, Habes M, Doshi J, Srinivasan D, Mamourian E, et al. (2020): Harmonization of large MRI datasets for the analysis of brain imaging patterns throughout the lifespan. Neuroimage 208:116450.
27. [preprint] Pedregosa F, Varoquaux G, Gramfort A, Michel V, Thirion B, Grisel O, et al. (2018): Scikit-learn: machine learning in Python. arXiv. Available from: http://arxiv.org/abs/1201.0490

28. [preprint] Paszke A, Gross S, Massa F, Lerer A, Bradbury J, Chanan G, et al. (2019): PyTorch: an imperative style, high-performance deep learning library. arXiv. Available from: http://arxiv.org/abs/1912.01703
